# A molecularly distinct cell type in the midbrain regulates intermale aggression behaviors in mice

**DOI:** 10.7150/thno.101658

**Published:** 2025-01-01

**Authors:** Chunyang Li, Cheng Miao, Yao Ge, Jiaxing Wu, Panpan Gao, Songlin Yin, Pei Zhang, Hongbin Yang, Bo Tian, Wenqiang Chen, Xiao Qian Chen

**Affiliations:** 1 Institute of Trauma and Metabolism of Zhengzhou University, Zhengzhou Central Hospital Affiliated to Zhengzhou University, Zhengzhou 450007, China.; 2 Department of Pathophysiology, School of Basic Medicine, Tongji Medical College, Huazhong University of Science and Technology, Wuhan 430030, China.; 3 Key Laboratory of Neurological Diseases, Ministry of Education; Hubei Provincial Key Laboratory of Neurological Diseases, Wuhan, Hubei Province 430030, China.; 4 Department of Neurobiology, School of Basic Medicine, Tongji Medical College, Huazhong University of Science and Technology, Wuhan 430030, China.; 5 Key Laboratory of Developmental Genes and Human Disease, School of Life Science and Technology. Southeast University, Nanjing 210096, China.; 6 Department of Affiliated Mental Health Center of Hangzhou Seventh People's Hospital, Liangzhu Laboratory, The State Key Lab of Brain-Machine Intelligence, Zhejiang University, Hangzhou 310000, China.; 7 MOE Frontier Science Center for Brain Science & Brain-Machine Integration, School of Brain Science and Brain Medicine, Zhejiang University, Hangzhou 310000, China.; 8 NHC and CAMS Key Laboratory of Medical Neurobiology, Zhejiang University, Hangzhou 310000, China.; 9 Section of Integrative Physiology and Metabolism, Joslin Diabetes Center and Department of Medicine, Harvard Medical School, Boston, MA 02215, USA.; 10 Translational Medicine, Steno Diabetes Center Copenhagen, Herlev 2730, Denmark.

**Keywords:** Periaqueductal gray, Tachykinin, Aggression, Translating ribosome affinity purification

## Abstract

**Rationale**: The periaqueductal gray (PAG) is a central hub for the regulation of aggression, whereas the circuitry and molecular mechanisms underlying this regulation remain uncharacterized. In this study, we investigate the role of a distinct cell type, *Tachykinin 2*-expressing (Tac2^+^) neurons, located in the dorsomedial PAG (dmPAG) and their modulation of aggressive behavior in mice.

**Methods**: We combined activity mapping, *in vivo* Ca^2+^ recording, chemogenetic and pharmacological manipulation, and a viral-based translating ribosome affinity purification (TRAP) profiling using a mouse resident-intruder model.

**Results**: We revealed that dmPAG^Tac2^ neurons are selectively activated by fighting behaviors. Chemogenetic activation of these neurons evoked fighting behaviors, while inhibition or genetic ablation of dmPAG^Tac2^ neurons attenuated fighting behaviors. TRAP profiling of dmPAG^Tac2^ neurons revealed an enrichment of serotonin-associated transcripts in response to fighting behaviors. Finally, we validated these effects by selectively administering pharmacological agents to the dmPAG, reversing the behavioral outcomes induced by chemogenetic manipulation.

**Conclusions**: We identify dmPAG^Tac2^ neurons as critical modulators of aggressive behavior in mouse and thus suggest a distinct molecular target for the treatment of exacerbated aggressive behaviors in populations that exhibit high-level of violence.

## Introduction

The periaqueductal gray (PAG) is a conserved brain region located in the midbrain traditionally recognized as a center integration hub for processing upstream emotion-related information 1-3. Early studies demonstrated that electrical stimulation of the PAG in cats could directly elicit an aggressive-defensive response, whereas lesions in the PAG abolished this response 4, 5. Although electrical stimulation of the amygdala and hypothalamus can also induce aggressive responses, PAG stimulation can still elicit robust aversive responses even then the amygdala and hypothalamus are lesioned 6, suggesting that the PAG has an independent role in eliciting aggressive responses.

Functional activity-dependent studies also confirmed the critical roles of the PAG in modulating aggression and defense behaviors. More recent studies using optogenetics, Ca^2+^ imaging, and electrophysiological recording have revealed causal links between PAG activity and aggressive/defensive behaviors. Notably, many of these studies have focused on subregions such as the dorsal PAG (dPAG) and the lateral PAG (lPAG) in a pan-neuronal context 7, 8. However, it is not well understood whether there is an anatomically and molecularly distinct cell type within the PAG that regulates aggressive behaviors.

In recent years, the crucial role of Tac2 positive (Tac2^+^) neurons in emotional behaviors such as fear memory and social behaviors has been demonstrated 9, 10, whereas most of these studies have focused on Tac2^+^ neurons within the limbic systems such as the amygdala and the hypothalamus, and the potential role of Tac2^+^ neurons in the PAG remains unexplored. In this study, we focused on Tac2^+^ neurons in the midbrain, specifically in the dorsomedial part of the PAG (dmPAG). Using a resident-intruder (RI) paradigm in mice, we revealed a critical role for dmPAG^Tac2^ neurons in intermale aggression. We further explored potential molecular targets in dmPAG^Tac2^ neurons through TRAP-seq profiling and pharmacological validation. Collectively, these findings indicate that the *Tac2*-expressing neurons serve as a molecularly distinct cell population within the PAG that regulates intermale aggression, suggesting that targeting these neurons might help alleviate symptoms in humans exhibiting high levels of aggression and violence.

## Methods

### Animals

Adult (8-10 weeks) *Tac2*-Cre mice (Jackson Laboratory, B6.129-Tac2tm1.1(cre)Qima/J, Strain #: 018938), Ai9-tdTomato reporter mice (Jackson Laboratory, B6.Cg-Gt(ROSA)26Sortm9(CAG-tdTomato)Hze/J, Strain #: 7909), and wild-type (WT) mice were used in this study. *Tac2*-Cre mice were generously provided by Prof. Minmin Luo from the Chinese Institute for Brain Research, Beijing, China and Ai9-tdTomato reporter mice by Prof. Jian-Zhi Wang from Huazhong University of Science and Technology (HUST). All mice were maintained on a C57BL/6J background under a 12h/12h light-dark cycle with food and water provided *ad libitum*. All procedures were conducted according to institutional guidelines and approved by the Animal Care and Use Committee at the HUST.

### Viruses

The viral vectors AAV_2/9_-hSyn-DIO-mCherry and AAV_2/5_-EF1a-DIO-DTA-mCherry were acquired from BrainVTA Co., Ltd (Wuhan, China). The viral vectors AAV_2/9_-hSyn-GCaMP6m, AAV_2/9_-hSyn-DIO-GCaMP6m, AAV_2/9_-hSyn-DIO-hM3Dq-mCherry, AAV_2/9_-hSyn-DIO-hM4Di-mCherry, and AAV_2/9_-hSyn-FasDO-GCaMP6m were prepared by Shanghai Taitool Bioscience Co., Ltd (Shanghai, China). The viral vector AAV_2/5_-FLEX-EGFPL10a was purchased from Addgene (Watertown, USA). All viruses were aliquoted and stored at -80℃ until use, with titers and volumes described in the corresponding method descriptions below.

### Stereotaxic surgeries and viral injections

All stereotaxic surgeries were performed under general anesthesia using a stereotaxic apparatus (Stoelting, USA). Mice were anaesthetized via intraperitoneal injection (i.p.) of chloral hydrate (350 mg/kg) and xylazine (10 mg/kg) before placed on a stereotactic apparatus (RWD Life Science, China), with a heating pad attached to maintain a stable body temperature. During the surgery, mice were eye-protected with ointment. After the skull was exposed and leveled on the stereotactic apparatus, the dmPAG (AP: -4.48 mm, ML: -0.03 mm, DV: -2.0 mm) was targeted for viral injection using calibrated glass microelectrodes connected to an infusion pump (SYS-Micro4, World Precision Instruments, UK) at a rate of 30 nl/minute. The injection needle was held still in place for 10 minutes before withdrawal, after which the incision was sutured. Mice were kept on a heating pad until fully recovered from anesthesia.

### *In vivo* fiber photometry recording

*In vivo* fiber photometry recording was performed as previously described 11, 12. AAV-hSyn-GCaMP6m (1.1×10^13^ vg/ml, 400 nl) or AAV-hSyn-DIO-GCaMP6m (2.4×10^13^ vg/ml, 200 nl) was injected into the dmPAG. After recovering for a period of two weeks, a unilateral implantation of optical fiber (200 µm, NA = 0.37; Inper LLC, Hangzhou, China) was performed on mice, which was about 200 μm above the dmPAG (AP: -4.48 mm, ML: -0.03 mm, DV: -1.8 mm). Following optical fiber implantation, mice were allowed to recover for at least 7 days before the RI assay. During the 15-minute test, behavioral performance and calcium activity of dmPAG neurons were recorded. We used 405 nm LED and a 470 nm LED, with the 405 nm LED serving as an isosbestic control for motion artifacts and fluorescence changes unrelated to neuronal activity, as previously reported 13. The 470 nm fluorescence was used to record Ca^2+^-sensitive GCaMP6m signal. Raw 405 nm and 470 nm fluorescence signals were processed using a polynomial fit to generate corrected signals, respectively, which were subsequently converted to normalized fluorescence (*F*) or z-scored traces.

To explore activity of dmPAG^Tac2^ neurons during various stimuli, we recorded GCaMP6m fluorescence intensity when these mice were subjected to the RI test, object exploration, exposure to fox odor, and pain stimulation, respectively. Raw Ca^2+^ fluorescence data (*F*) were collected by fiber photometry (sampling frequency at 25 Hz) and normalized using baseline correction, motion correction, and signaling filtering using Inper Data Process Software (Inper LLC, China). The Ca^2+^ fluorescence was aligned to the onset of corresponding behavioral events, and the change in fluorescence (Δ*F/F*) were calculated as the formula (*F*-*F_0_*)/*F_0_*, where *F* is normalized fluorescence and *F_0_* represents the baseline fluorescence signal averaged over a 4-second time window, from -4 seconds to 0 seconds prior to the onset of the events. Data processing, analysis and visualization were performed in MATLAB (R2017a) and GraphPad Prism (v8).

### Behavioral assays

Mice were acclimated to the testing room for at least 3 hours prior to each test to habituate to the environments. After each testing trial, all behavioral apparatus were cleaned with 75% ethanol to eliminate the residual odor from previous mice. All behavioral tests were performed during the dark phase of the mice (1 hour after lights were switched off) under dim red light.

### Open-field test (OFT)

The OFT was performed as described previously with minor modifications 12, 14, 15. Briefly, a white open-field box (50 cm × 50 cm × 50 cm) made of PVC materials was used. Mice were placed gently in the center of the arena (a starting position) and allowed to explore freely for 10 minutes. Data were recorded and analyzed using SuperMaze software (Shanghai Xinruan Informatlon Technology Co.Ltd, China). The total distance travelled, time spent in the central zone, and total numbers of entries into the central zone were measured.

### Object exploration test

The object exploration was performed as previously described with minor modifications 16. Using the same open-field arena as the OFT test, mice were initially allowed to adapt to an empty arena. Then they were placed in the arena containing an inanimate object for 5 minutes, during this period, both behavioral performance and Ca^2+^ fluorescence of dmPAG^Tac2^ neurons were recorded. The recording was aligned to the onset of exploratory behavior, which was defined as when the mouse directed its nose toward the object within a 2-cm distance.

### Elevated-plus maze (EPM) test

The EPM test was performed as previously reported with minor modifications 12, 14, 15. Briefly, an EPM apparatus was placed 62-cm above the ground, which was comprised of two open arms (75 cm × 5 cm) and two closed arms (75 cm × 5 cm × 15 cm) (Shanghai Xinruan Informatlon Technology Co.Ltd, China). To begin with the test, mice were placed in the center area of the maze, facing towards an open arm. Their behaviors were recorded for 8 minutes using SuperMaze software. The time spent in open or closed arms and the numbers of entries into each arms were analyzed.

### Rotarod test

To examine muscle strength and motor coordination, we performed the rotarod test as previously described with minor modifications 12, 14, 15. Prior to the test, mice received a low-speed training (accelerated from 5 to 10 rpm) in three daily sessions, each lasted for 30 minutes with a 30-minute interval. During the test over a 5-minute period, the speed was accelerated from 5 to 40 rpm. Each mouse was tested for four trials per day, with a 30-minute interval between trials. The latency to fall was automatically recorded and analyzed.

### Three-chamber social interaction test

The three-chamber social interaction test was conducted in a rectangular plexiglass box (60 cm × 40 cm × 22 cm), as previously reported 12, 14. The box was divided into three equal chambers, separated by two clear plexiglass walls, each containing an entryway allowing for free movement between chambers. A wire cage (5 cm in diameter and 10 cm in height) was placed in the center of each side chambers. To start the test, mouse was placed in the middle chamber. During Session #1 (habituation), empty wire cages were placed in both side chambers. The mouse was allowed to freely explore for 10 minutes. During Session #2 (sociability test), a stranger mouse of the same species and sex was placed inside one of the wire cages, and the testing mouse was again allowed to explore for 10 minutes. During Session #3 (social novelty test), a new stranger mouse of the same species and sex but from a different cage than the first mouse placed in Session #2. The testing mouse was then allowed to explore for another 10 minutes. Between each session, there was a 5-minute interval. Data were recorded and analyzed using SuperMaze software. The social preference score was calculated as (*T*_S_ -* T*_NS_)* /* (*T*_S_ +* T*_NS_), in which *T*_S_ or *T*_NS_ represent the time a testing mouse spent in social or non-social area, respectively.

### 2-methyl-2-thiazoline (2MT) odor stimuli

The 2MT odor stimuli assay was conducted as previously described 17-19. For fiber photometry recordings of dmPAG^Tac2^ neuron responses to fear stimuli, 2-methyl-2-thiazoline (2MT), a potent analog of the fox odor 2,4,5-trimethyl-3-thiazoline (TMT), was used to elicit innate fear or defensive behaviors such as freezing. Each mouse was recorded in its home cage while single-housed in the testing room. Following a 5-minute exposure to a control odor (saline), 20 μl of 2MT (2.1 × 10^-4^ mole) was applied to a piece of filter paper placed in a small dish in the corner farthest from the mouse. Behavioral performances and calcium activity of dmPAG^Tac2^ neurons were recorded under saline and 2MT exposure.

### Hot plate pain test

The hot plate pain test was performed as described 17, 20. Mice were placed on a metal plate (275 mm × 263 mm × 15 mm) for an initial period of 5-minute acclimation at 30°C. The temperature of the plate was increased at a rate of 6 ℃ per minute, to a maximum of 55 °C until the mouse manifested aversive behaviors such as hind-paw licking, shaking, lifting, or jumping. Nociceptive responses to heat and calcium activity were recorded for analysis.

### Resident-intruder (RI) test

The RI test is used to assess aggressive behaviors in mice 21, though specific protocols can vary depending on research focus and objectives 22. In our study, “resident” mice used for the RI test were housed with a female companion for at least 7 days prior to the test, which could enhance territorial behavior. During this period, the bedding material was maintained unchanged. We avoided choosing the commonly used aggression-increasing protocol of prolonged single housing prior to the RI test, as previous studies have shown that chronic social isolation broadly upregulates Tac2/NkB signaling across brain regions 10. One hour before RI test, the female companion mouse was removed, and a male C57BL/6J “intruder” mouse, slightly smaller in body size than the resident, was introduced into the cage for a 15-minute interaction period. An effective attack was defined by behaviors such as biting, wrestling, or chasing exhibited by the resident towards the intruder 23-25. Other behaviors, including sniffing (defined as nose contact with any part of the intruder's body, including genital sniff) 26, and social grooming 27, were also recorded and analyzed. Latency to attack, number of attack episodes, and duration of attacks were analyzed using Adobe Premiere Pro CC 2018 and GraphPad Prism 8.0.

### Chemogenetic modulation of dmPAG^Tac2^ neurons

Chemogenetic manipulation with clozapine-N-oxide (CNO, C0832, Sigma-Aldrich, i.p.) was performed as previously reported 12, 14, 15. Based on previous reports 11, 25, 26, 28, 29, we chose a CNO dosage of 0.5 mg/kg for chemogenetic activation and 1.0 mg/kg for chemogenetic inhibition, selected from a testing range of 0.1 to 0.7 mg/kg for chemogenetic activation and 0.5 to 7.5 mg/kg for chemogenetic inhibition, respectively. To activate dmPAG^Tac2^ neurons, *Tac2*-Cre mice were injected with AAV-hSyn-DIO-hM3Dq-mChery (Gq, 1.22×10^13^ vg/ml, 200 nl) into the dmPAG (*Tac2*^hM3Dq^ mice), while control mice (*Tac2*^mCherry^ mice) received AAV-hSyn-DIO-mChery (5×10^12^ vg/ml, 500 nl). To avoid a potential “ceiling effect”, all mice pre-screened twice to select individuals with low baseline levels of spontaneous attacks 26. Twenty-one days after virus injections, mice were tested for aggression for three consecutive days to confirm low levels of spontaneous attacks. Mice then received either CNO (0.5 mg/kg) or normal saline (control) and 30 minutes prior to the RI test. The efficacy of CNO-activated Gq virus in dmPAG^Tac2^ neurons was verified by c-Fos staining 120 minutes after CNO administration in normal home-cage housed* Tac2*^hM3Dq^ mice.

For chemogenetic inhibition of dmPAG^Tac2^ neurons, *Tac2*-Cre mice with high baseline levels of spontaneous attacks were pre-selected prior to virus injection to avoid a potential “flooring effect” 26. Eligible mice received injection of either AAV2-hSyn-DIO-hM4Di-mChery (Gi, 1.77×10^13^ vg/ml, 150 nl, *Tac2*^hM4Di^) or AAV2-hSyn-DIO-mChery (*Tac2*^mCherry^). Twenty-one days after viral injection, mice were tested for higher spontaneous attacks over three consecutive days. *Tac2*^hM4Di^ and *Tac2*^mCherry^ mice were then administrated with CNO (1.0 mg/kg) or normal saline 30 minutes prior to the RI test.

### Genetic ablation of dmPAG^Tac2^ neurons

*Tac2*-Cre mice with high baseline levels of spontaneous attacks were pre-selected for genetic ablation experiment. To ablate dmPAG^Tac2^ neurons, AAV-hSyn-DIO-DTA-mCherry (5.89×10^12^ vg/ml, 450 nl) was injected into the dmPAG of *Tac2*-Cre mice, while control mice received AAV-hSyn-DIO-mCherry. Twenty-one days after viral injection, mice were subjected to the RI test.

### Immunofluorescence

Immunofluorescent staining was performed as previously described 30, 31. Mice were perfused with ice-cold normal saline and subsequently 4% paraformaldehyde (PFA). After perfusion, whole brains were removed from the skull and post-fixed in 4% PFA (6 hours, 4°C), and transferred to 20% and 30% sucrose solutions for gradient dehydration.

To examine Tac2 expression, brains from *Tac2*-Cre/Ai9-tdT mice were used (with tdTomato^+^ in Tac2^+^ neurons). After dehydration, 30-μm coronal sections were cut from frozen brain tissues using a vibratome (Leica 1860). Brain slices were rinsed in phosphate-buffered saline (PBS) for three times (5 minutes each) and stained with Hoechst (1:1000, #33342, Sigma-Aldrich) for 10 minutes, and then were cover-slipped with fluorescent mounting medium and imaged immediately.

To verify immunofluorescent expression and viral injection site, brains that underwent stereotaxic surgeries were used. Coronal sections containing the PAG were cut at a thickness of 20 μm and then were rinsed in PBS three times, stained with Hoechst for 10 minutes. These slices were rinsed again in PBS three times, cover-slipped with fluorescent mounting medium and imaged immediately.

For c-Fos staining, we utilized a previously established protocol 32. Mice subjected to the RI test were sacrificed 90 minutes after the test or 120 minutes after receiving CNO administration. Coronal sections of the whole brain (for c-Fos mapping) or the PAG area (for CNO administration) were prepared at a thickness of 20 μm. Brain slices were permeabilized with 0.3% Triton X-100 in PBS for 10 minutes and blocked with 5% goat serum in PBS for 1 hour. Slices were incubated with primary antibodies (1:800, rabbit monoclonal anti-cFos antibody, #2250S, Cell Signaling) at 4°C overnight, followed by three PBS rinse and incubation with a fluorochrome-conjugated secondary antibody (1:200, Dylight^488^-conjugated goat anti-rabbit, Abbkine; 1:200, AF^488^-conjugated goat anti-rabbit IgG, Jackson ImmunoResearch) for 1 hour at room temperature in the dark. After final PBS rinses, nuclei staining with Hoechst was performed for 10 minutes, rinsed again, cover-slipped with fluorescent mounting medium and stored in 4°C until imaging.

### Translating ribosome affinity purification (TRAP) and RNA sequencing

Affinity purification of translating ribosomes was performed as described with minor modifications 33. *Tac2*-Cre mice received stereotaxic injection of AAV-EF1a-FLEX-EGFPL10a (7×10^12^ vg/ml, 400 nl) in the dmPAG. Three weeks later, brain areas containing the dmPAG were rapidly dissected (individual dmPAG pooled from 6-7 mice per sample; thus, 3 replicates per group) in the cold room (4°C) and immediately washed with ice-cold dissection buffer to remove blood. We used a set of pre-cooled douncer-based method containing a loose pestle douncer and a tight pestle douncer (Kimble 885300-0002). These samples were transferred to a pre-chilled homogenizer placed on ice containing tissue-lysis buffer (20 mM HEPES, pH 7.4, 10 mM MgCl_2_, 150 mM KCl, 0.5 mM DTT, 100 µg/ml cycloheximide, 400U/ml RNasin and 200U/ml Superasin). Tissues were homogenized for initially in a loose pestle douncer and then in a tight pestle douncer. Homogenates were centrifuged at 2,000 g for 10 minutes at 4°C to pellet large debris. NP-40 (1%) and 1,2-diheptanoyl-sn-glycero-3-phosphocholine (30 mM, Avanti Polar Lipids, AL) were added to the supernatant, incubated on ice for 5 minutes, then centrifuged at 20,000 g for 10 minutes to remove insoluble materials. A portion of the supernatant (~5%) was collected as “Input” fraction. GFP antibodies (5 μg, Proteintech, USA) were added into the remaining supernatant and incubated with end-over-end rotation for 4 hours at 4°C. During this time, protein A+G magnetic beads (Beyotime Biotechnology, China) were prepared. Cleaned beads were added to the homogenates after initial incubation was finished, and the tissue homogenates were incubated with end-over-end rotation for an additional 16-18 hours at 4°C. Beads were then washed four times with high-salt buffer (20 mM HEPES KOH, pH 7.3, 350 mM KCl, 10 mM MgCl_2_, 1% NP-40, 0.5 mM DTT, 100 μg/ml cycloheximide, and 400U/ml RNasin). After the final wash, pre-cooled centrifuge tubes were placed on a magnet holder for at least 1 minute, and residual wash buffer was removed. Afterwards, RLT buffer (Qiagen Kit 74004, with DTT) was added, tubes were removed from the magnet holder and vortexed for 30 seconds at room temperature. After placing the tubes back on the magnet holder for at least 1 minute, the RLT buffer containing RNA was collected as “IP” fraction. RLT buffer was also added to the Input samples for RNA extraction, which was performed side-by-side with the IP samples according to the manufacturer's instructions. Finally, extracted RNAs or RLT lysate containing RNA was sent to Shanghai Bohao Biotechnology for RNA quality inspection and subsequent library construction and mRNA sequencing.

### Intra-dmPAG drug delivery for pharmacological validation

Pharmacological validation were performed based on previous reports with minor modifications 9, 10, 34-41. First, AAV2-hSyn-DIO-hM3Dq-mCherry or AAV2-hSyn-DIO-hM4Di-mChery was injected into the dmPAG of *Tac2*-Cre mice. Two weeks later, these mice were implanted with a guide cannula in the dmPAG (AP: -4.48 mm, ML: -0.03 mm). The depth of the injection catheter base was determined based on the target injection site in the brain and the corresponding injection tube depth. For instance, the injection site of the dmPAG of a 25g mouse in this study was -2.0 mm. If the implantation depth of the catheter base was -1.40 mm, a 0.6 mm injection tube (G1) was used. The implantation depth for each mouse was recorded to ensure the correct injection tube length was used in subsequent drug administration experiments. A catheter cap was inserted into the drug delivery catheter between injections to maintain patency, prevent thrombosis, and reduce the risk of intracranial infection.

Seven days after cannula implantation, pharmacological agents, fluoxetine (6 µg/500nl, MCE), CP-93129 (1 µg/500nl, MCE), SB-224289 (1 µg/500nl, MCE), or vehicle (saline with DMSO) was delivered into the dmPAG. Before behavioral testing, the guide cannula (RWD Life Science, China) was removed and replaced with an injection cannula, which protruded 0.5-0.8 mm beyond the tip of the guide cannula, based on the guide base implantation depth. The injection cannula was connected to a 10-µl Hamilton syringe with PE tubing. Drug solutions were administered using an injection pump at a rate of 0.25 µl/min to a total volume of 500 nl. After drug delivery, the injection cannula was held in place for 3 minutes to allow diffusion and to prevent backflow upon withdrawal. The injection cannula was then slowly removed, and the remaining drug in the PE tube was expelled to verify needle patency. After the intracranial injection, CNO was i.p. administered immediately. The guide cannula was sterilized and reinserted into the guide base. Behavioral testing was performed approximately 30 minutes after CNO administration.

### Quantification and statistical analysis

Data were processed and analyzed using various softwares. Statistical analyses included unpaired *t*-test, paired *t*-test, one-way ANOVA and two-way ANOVA for data with homogeneity of variance and normal distribution. Tukey's test was used for multi-comparisons between groups following ANOVA. Data are expressed as mean ± S.E.M. Statistical significance was indicated with *P < 0.05, **P < 0.01, ***P < 0.001, ****P < 0.0001. Specifically, one-way ANOVA followed by Tukey's multiple comparisons test analysis was used to compare fluorescent staining in different brain region. Paired *t*-test was applied to chemogenetic manipulation and intra-dmPAG drug microinfusion 11. Unpaired *t*-test was used to compare behavioral results of WT and *Tac2*-Cre mice in the open-field, elevated plus maze, and rotarod test. Two-way ANOVA followed by Tukey's multiple comparisons test was used for analyzing data from the three-chamber social interaction test, and repeated measures two-way ANOVA was used to analyze behavioral data for ablation of dmPAG^Tac2^ neurons. Behavior data from the RI test were collected by Xinsoft XR-XZ301 video acquisition and analysis system, with video analysis and statistics performed by Adobe Premiere Pro CC 2018. A double-blind analysis of all the videos was performed by at least two independent investigators unaware of the group information. The mean of the two independently collected datasets was used for further statistical analysis.

## Results

### *Tac2*-expressing neurons exhibit distinct expression pattern in the PAG

**Figure 1:**
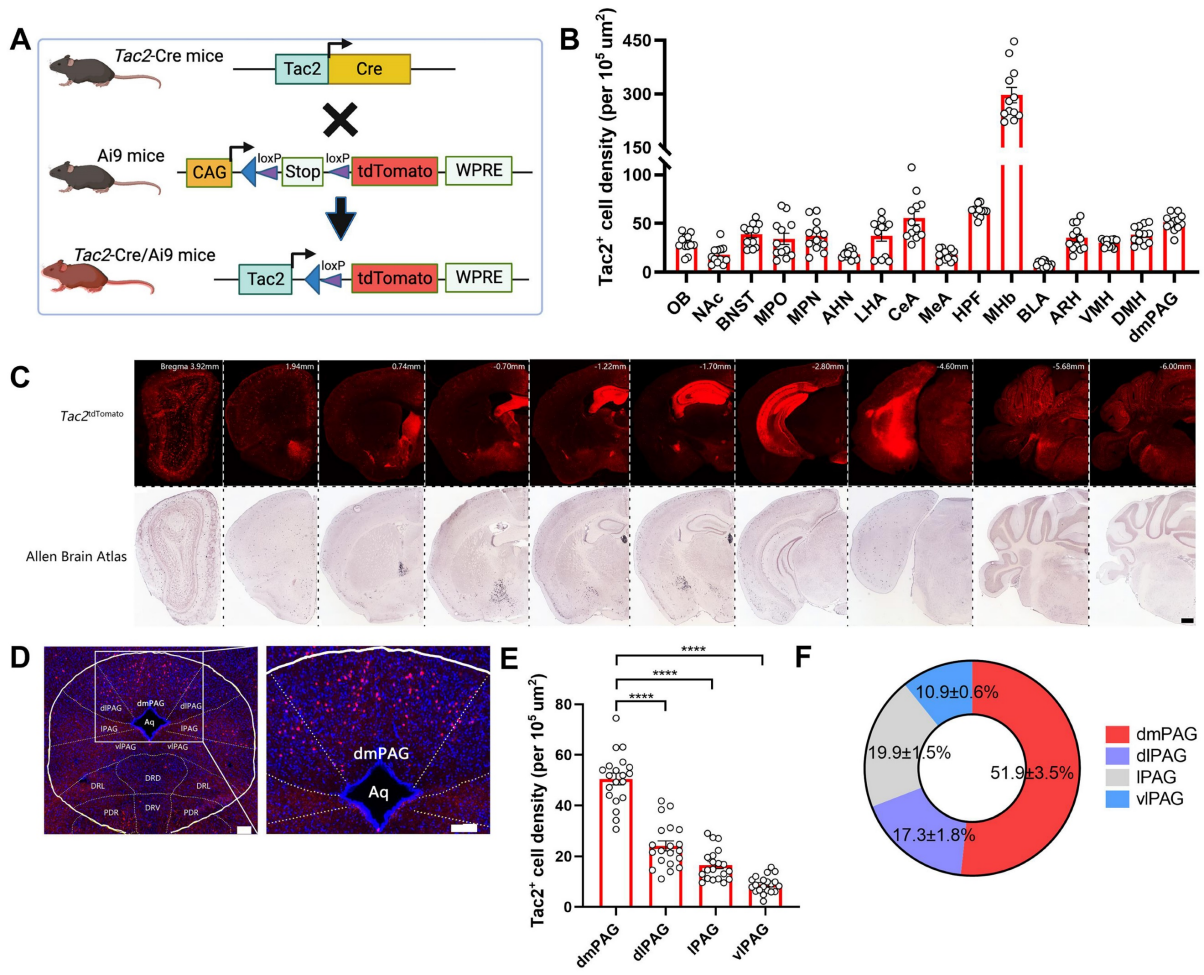
** PAG *Tac2*-expressing neurons are mainly located in the dmPAG. (A)** Schematic showing breeding strategy to selectively express tdTomato reporter in Tac2^+^ cells. **(B)** Whole-brain analysis of *Tac2*^tdTomato^ cell density, showing high expression levels in the MHb, HPF, dmPAG. N=3 mice, with 4 brain sections from each mouse. **(C)** Representative fluorescent images showing tdTomato-labeled Tac2^+^ cells (red) in coronal brain sections in *Tac2*-Cre/Ai9 mice (upper panels) and in their respective ABA images (lower panels). Scale bar, 500 μm. **(D)** Distribution pattern of Tac2^+^ cells in the PAG subregions. Scale bars, 1,000 μm (left) and 100 μm (right, enlarged view), respectively. **(E)** Analysis of *Tac2*^tdTomato^ cells in PAG subregions. N=5 mice, with 4 brain sections from each mouse. **(F)** Pie chart showing percentage of *Tac2*^tdTomato^ cells in PAG subregions. A total of 648 Tac2^+^ cells were included from N = 3 mice. Data were expressed as mean ± S.E.M. Significance was calculated by One-way ANOVA and Tukey's multiple comparisons test, ****P < 0.0001. *Abbreviations*: OB: Olfactory bulb; NAc: nucleus accumbens; BNST: bed nuclei of the stria terminalis; MPO: medial preoptic area; MPN: medial preoptic nucleus; AHN: anterior hypothalamic nucleus; LHA: lateral hypothalamic area; CeA: central amygdalar nucleus; MeA: medial amygdalar nucleus; HPF: hippocampal formation; MHb: medial habenula; BLA: basolateral amygdalar nucleus; ARH: arcuate hypothalamic nucleus; VMH: ventromedial hypothalamic nucleus; DMH: dorsomedial nucleus of the hypothalamus; dmPAG: dorsomedial periaqueductal gray.

Previous studies have reported that Tac2^+^ neurons are expressed across various brain regions, including the amygdala, hypothalamus, and nucleus accumbens 42. However, their expression pattern within the PAG has not been fully characterized. To this end, we crossed *Tac2*-Cre knock-in mice with Ai9 reporter mice, which enables selective expression of tdTomato fluorescent protein in Tac2^+^ cells (Figure 1A). By aligning the expression of *Tac2*^tdTomato^ cells with the Allen Brain Atlas (ABA), we confirmed that *Tac2*^tdTomato^ cells were located in specific brain regions such as the amygdala, hypothalamus, and habenula (Figure 1B-C), consistent with previous reports 10, 42. We note that the medial habenula (MHb) has the highest cell density compared to other brain regions, and Tac2 has been suggested as a marker for the MHb 43. No tdTomato^+^ cells were detected in control mice, including *Tac2*-Cre, Ai9, and wide-type (WT) mice (Figure S1A).

We then specifically examined the expression pattern within the PAG. *Tac2*^tdTomato^ cells were expressed throughout the PAG (Figure 1D), consistent with the mRNA expression pattern noted in the ABA (Figure S1B). However, *Tac2*^tdTomato^ cells were particularly enriched in the dmPAG (Figure 1E-F), comprising approximately 50% of the total *Tac2*-expressing cells in the pan-PAG areas. This distribution closely resembles the enriched distribution of *Tac2* mRNAs in the dmPAG, accounting for 54.4% of Tac2 mRNA signals (Figure S1C-D). Thus, these findings indicate that *Tac2*-expressing cells in the PAG are predominantly located in the dorsomedial subregion.

### dmPAG^Tac2^ neurons respond to aggression

**Figure 2:**
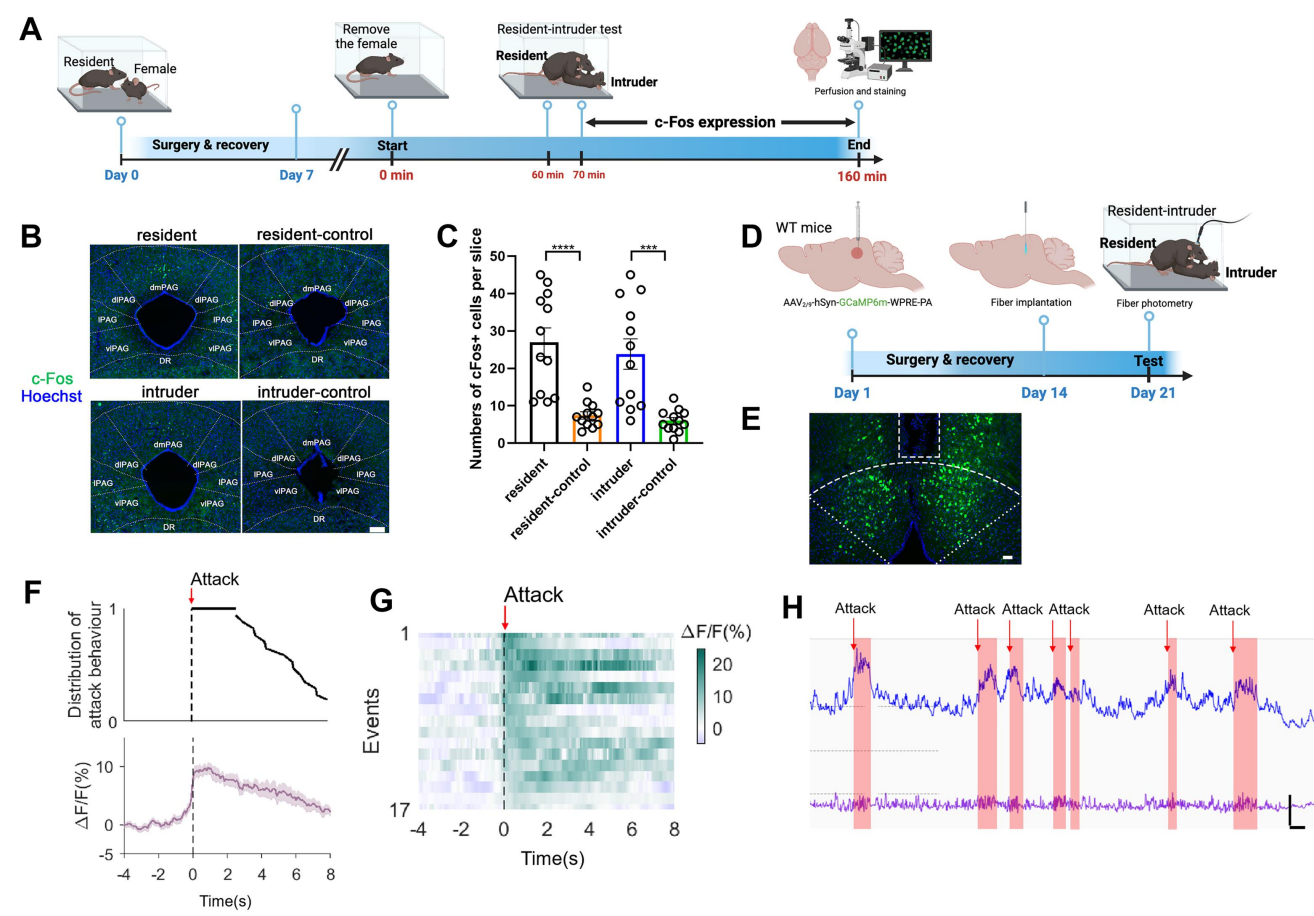
** dmPAG neurons respond to aggression. (A)** Strategy for mapping c-Fos expression in WT mice subjected to the RI test. **(B)** c-Fos activation in dmPAG of mice from various groups. Scale bar, 100 μm. **(C)** Number of c-Fos^+^ cells in the dmPAG of WT mice. N = 3 mice/group, with 4 brain sections from each mouse. **(D)** Strategy for *in vivo* fiber photometry in WT mice injected with AAV-hSyn-GCaMP6m.** (E)** Fiber position targeting the dmPAG of WT mice. Scale bar, 50 μm. **(F)** Distribution of attack behavior synchronized with Δ*F*/*F*%. **(G)** Heatmap showing Ca^2+^ activity time-locked to an attack episode. x-axis shows 4 sec prior to and 8 sec after the attack episode (red arrow). **(H)** Representative Ca^2+^ trace showing one trial of Ca^2+^ activity in a WT mouse during the RI test. Scale bar: 10s in x-axis and 2000 A.U. in y-axis. Baseline (bottom trace): reference control channel (405 nm); upper trace, Ca^2+^ channel (470 nm). Red shaded areas indicate attack episodes and durations. Data represent mean ± S.E.M. Significance was calculated by One-way ANOVA and Tukey's multiple comparisons test, ***P < 0.001, ****P < 0.0001.

We next sought to determine the involvement of dmPAG^Tac2^ neurons in fighting behaviors. First, we subjected the WT mice to the RI assay and harvested brain tissues following fighting episodes (Figure 2A). As expected, fighting behaviors activated neurons across multiple brain regions, including the paraventricular nucleus of the thalamus, periventricular zone, anterior hypothalamic nucleus (Figure S2). Focusing on c-Fos^+^ neurons in the dmPAG, our results showed a significant number in c-Fos^+^ neurons in the dmPAG of resident mice compared to the control mice maintained in home cages (Figure 2B-C). In contrast, fighting behaviors had minimal effects on c-Fos expression in other PAG subregions, such as the dlPAG, lPAG, and vlPAG (Figure S2B-C).

We further investigated the role of dmPAG neurons in response to fighting behaviors using *in vivo* fiber photometry. AAV expressing GCaMP6m was injected into the dmPAG of WT mice, followed by implantation of a fiber above the dmPAG (Figure 2D). Correct fiber placement was verified (Figure 2E). During the RI test, we observed a substantial increase in Ca^2+^ transients in response to each attack bout (Figure 2F-G). Notably, each attack elicited an immediate increase in Ca^2+^ activity at the onset of the attack episode, with Ca^2+^ levels returning to baseline following the attack (Figure 2H). These results demonstrate the involvement of dmPAG neurons in modulating fighting behaviors.

**Figure 3:**
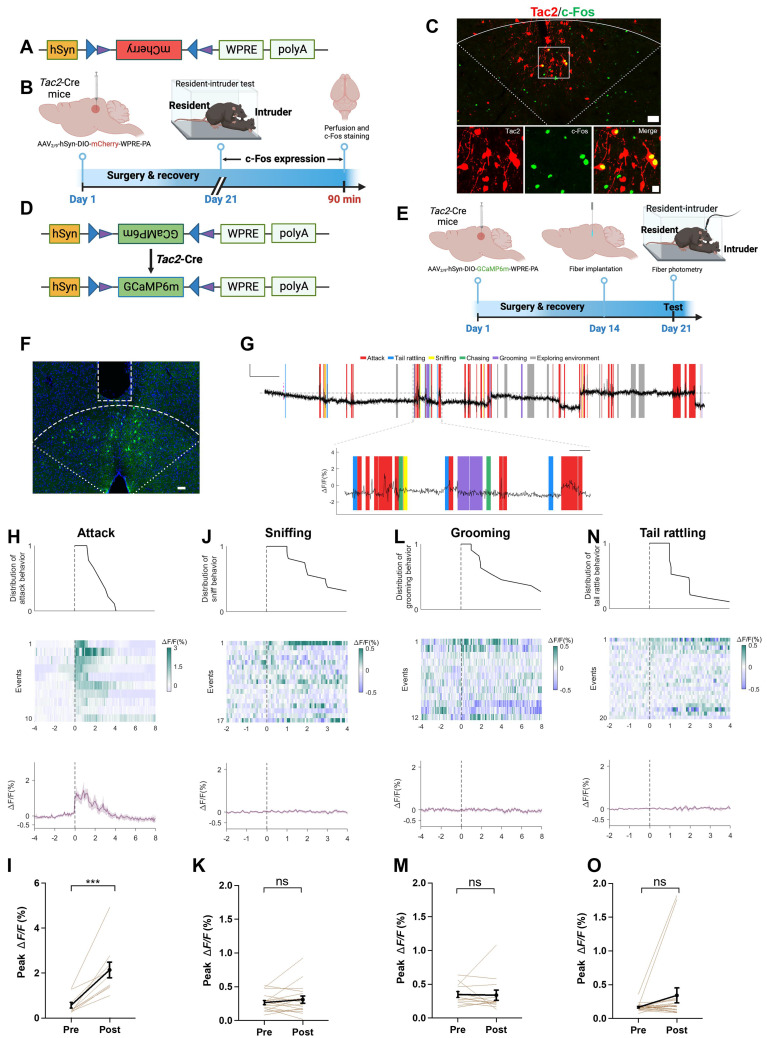
***Tac2*-expressing neurons in dmPAG respond to aggression. (A)** Vector information for AAV-hSyn-DIO-mCherry. **(B)** Strategy for examining activation of Tac2^+^ neuron by fighting behaviors. AAV vector was injected to *Tac2*-Cre mice to drive mCherry expression in dmPAG^Tac2^ neurons. **(C)** c-Fos expression (green) in dmPAG^Tac2^ neurons (red mCherry labeled) in *Tac2*-Cre mice subjected to the RI test. Lower panels showing enlarged view from the white box in the upper panel. Scale bars, 50 μm (upper), 20 μm (lower), respectively. **(D)** Vector information for AAV-hSyn-DIO-GCaMP6m in *Tac2*-Cre mice. **(E)** Strategy for *in vivo* fiber photometry in *Tac2*-Cre mice injected with Cre-dependent AAV-GCaMP6m vector. **(F)** Fiber position targeting dmPAG region of Tac2 mice. Scale bar, 50 μm. (G) Example recording of dmPAG^Tac2^ neurons during the RI test. Color shades indicate different behavioral syllables during intruder encounter. Scale bar: 60s in x-axis, 1.5% GCaMP6m Δ*F*/*F* signal in Y-axis. **(H-O)** Ca^2+^ activity time-locked to attack **(H-I),** sniffing **(J-K),** grooming **(L-M)** and tail rattling **(N-O)**. **(H)** Distribution of attack events (top), heatmap of GCaMP6m Δ*F*/*F* signals (middle) and Δ*F*/*F*% (bottom), **(I)** Analysis of peak Δ*F*/*F* before (Pre) and after (Post) each attack episode. The other panels present the same manner but with different behavioral syllables, including sniffing **(J-K)**, grooming **(L-M)**, and tail rattle **(N-O)**. Data represent mean ± S.E.M. Paired *t*-test, ***P < 0.001; ns, no significance.

We further investigated the role of dmPAG^Tac2^ neurons in fighting behaviors. To selectively label dmPAG^Tac2^ neurons, we used a Cre-dependent AAV vector (Figure 3A) and subsequently subjected the mice to the RI assay (Figure 3B). Immunostaining for c-Fos proteins in mCherry^+^ cells confirmed that dmPAG^Tac2^ neurons were activated by fighting behaviors (Figure 3C). Next, we measured Ca^2+^ activity of dmPAG^Tac2^ neurons during the RI assay by injecting AAV carrying a Cre-dependent GCaMP6m vector (Figure 3D-E) and verified the correct placement of the optic fiber (Figure 3F). We observed an increase in Ca^2+^ activity in response to each attack episode (Figure 3G-I). When analyzing each component of an attack event in relation to Ca^2+^ activity (Figure 3G), we found that attacks reliably induced Ca^2+^ transients, whereas none of the other attack-associated behaviors, such as tail rattling, sniffing, and grooming, could significantly alter Ca^2+^ activity (Figure 3J-O).

Additionally, using a Cre-out strategy, we examined Tac2-negative neurons in the dmPAG and observed a mild but significant increase in Ca^2+^ activity following each attack episode (*P*<0.05, Figure S3A-E). This increase was, however, three times lower than observed in dmPAG^Tac2^ neurons (*P*<0.0001, Figure S3F), suggesting that while the other cell types in the dmPAG may contribute, at least partially, to fighting behaviors, the extent of Ca^2+^ activity increase (Figure 3H-I and Figure S3B-F) indicates a predominant role of dmPAG^Tac2^ neurons in response to aggression.

The PAG is a highly heterogenous region involved in various functions, including sensorimotor control and pain sensation 2, 44. To further assess the specificity of dmPAG^Tac2^ neurons in relation to fighting behaviors, we subjected these mice to a variety of stimuli and recorded Ca^2+^ activity. Results showed that dmPAG^Tac2^ neurons failed to exhibit significantly altered Ca^2+^ activity in response to male-male and male-female encounter (Figure S4A-F), rotarod activity (Figure S4G-H), object exploration (Figure S4I-J), fox odor sniffing (Figure S4K-L), saline sniffing (Figure S4M-N), or pain stimulation (Figure S4O-P). Collectively, these findings demonstrate that dmPAG^Tac2^ neurons respond specifically to fighting behaviors.

### Deletion or inhibition of dmPAG^Tac2^ neurons attenuates fighting behaviors

To further confirm the involvement of dmPAG^Tac2^ neurons in fighting behaviors, we performed chemogenetic manipulations to examine the causal role of these neurons in aggression. First, we performed a basic behavioral characterization of *Tac2*-Cre mice and their WT littermate controls. Results showed that *Tac2*-Cre mice exhibited comparable behavioral phenotypes to WT mice (Figure S5A-P). In addition, *Tac2*-Cre mice exhibited similar levels of aggression in the RI assay (Figure S5Q-T), establishing a non-different baseline level of aggression prior to our genetic ablation and chemogenetic manipulation experiments.

**Figure 4:**
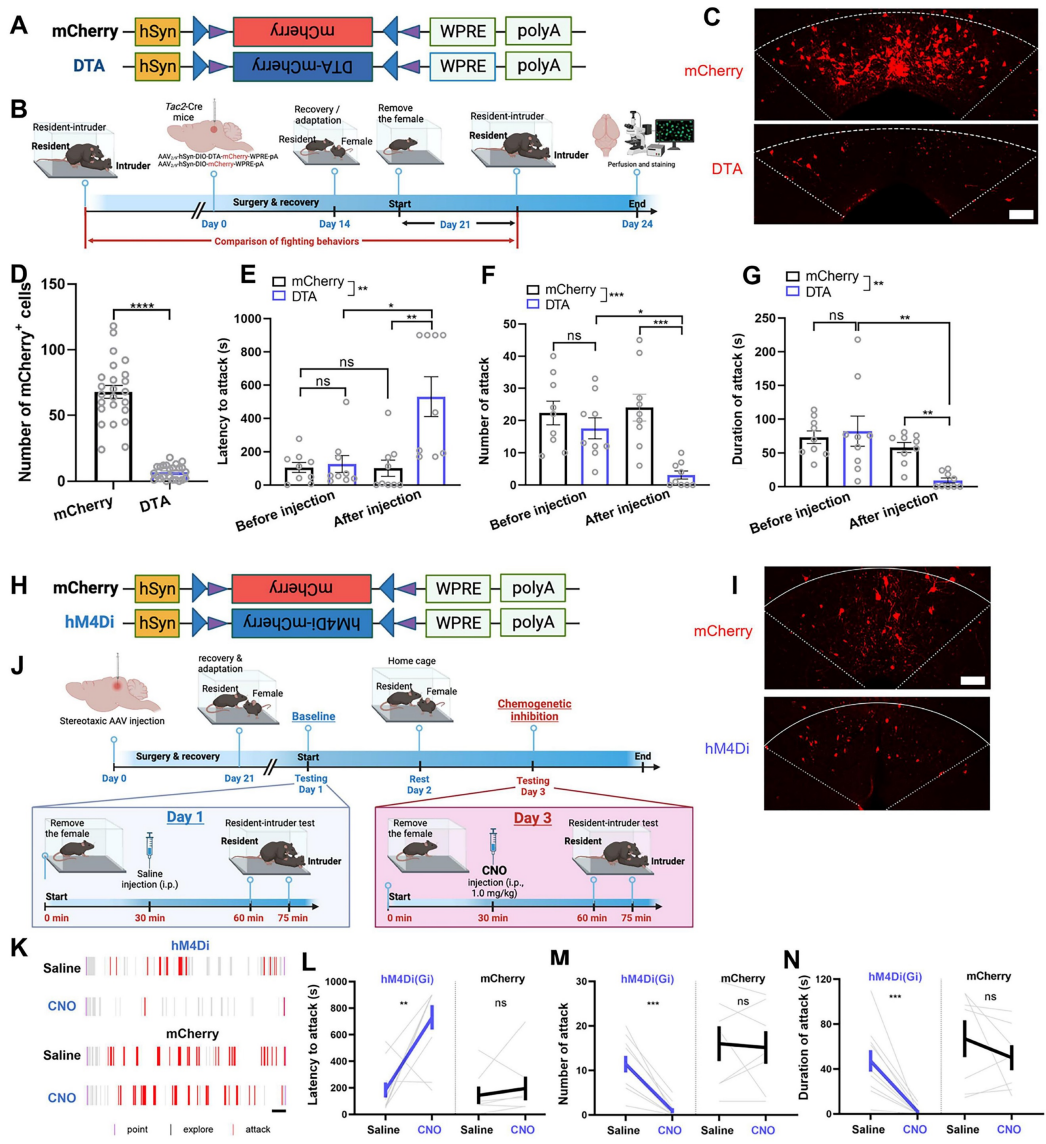
** Ablation or inhibition of dmPAG^Tac2^ neurons suppresses fighting behaviors. (A)** A Cre-dependent DTA vector or its control vector to be expressed in dmPAG^Tac2^ neurons. **(B)** Strategy for genetic ablation. **(C)** Validation of DTA (bottom) and control AAV (upper) in *Tac2*-Cre mouse. Scale bar, 100 μm. **(D)** Number of mCherry^+^ cells in dmPAG in *Tac2*-Cre mice receiving genetic ablation. N = 6 mice, with 4 brain sections from each mouse. **(E-G)** Analysis of latency to attack **(E)**, number of attack **(F)** and duration of attack **(G)**. N = 9/group. Significance was calculated with Two-way RM ANOVA followed by Tukey's multiple comparisons test. *P < 0.05, **P < 0.01, ***P < 0.001. **(H)** A Cre-dependent hM4Di vector or its control to be expressed in dmPAG^Tac2^ neurons. **(I)** Representative fluorescent images of mCherry (upper) or hM4Di-mCherry (bottom). Scale bar, 100 μm. **(J)** Strategy for the RI test in *Tac2*-Cre mice receiving inhibitory DREADDs vectors. **(K)** Raster plots showing mouse attack (red bar) or exploring behaviors (gray bar) during the RI assay. Scale, 1 minute. **(L-N)** Analysis of latency to attack **(L)**, number of attack **(M)** and duration of attack **(N)**. N = 10 in *Tac2*^hM4Di^ group and N = 7 in *Tac2*^mCherry^ group. Data represent mean ± S.E.M. Significance was calculated by means of paired *t*-test. **P < 0.01, ***P < 0.001; ns, no significance.

We then investigated the behavioral effects of genetic ablation of dmPAG^Tac2^ neurons by injecting a neuron-specific, Cre-dependent AAV encoding DTA-mCherry (Figure 4A-B) into the dmPAG of *Tac2*-Cre mice (referred to as *Tac2*^DTA^). *Tac2*-Cre mice injected with a Cre-dependent AAV-mCherry (Figure 4A-B) served as controls (referred to as *Tac2*^mCherry^). DTA-mediated ablation resulted in an 80% ablation of dmPAG^Tac2^ neurons (Figure 4C-D), confirming efficient ablation of *Tac2*-expressing cells in the dmPAG. *Tac2*^mCherry^ control mice showed consistent aggression levels before and after AAV injection; however, mice with genetic ablation of dmPAG^Tac2^ neurons exhibited significantly reduced aggression (Figure 4E-G), compared not only to their baseline levels but also to the *Tac2*^mCherry^ controls. Furthermore, ablation of dmPAG^Tac2^ neurons significantly decreased the percentage of mice exhibiting fighting behaviors (Figure S6A) without affecting male-male investigation during encounters (Figure S6B). These findings indicate that deletion of dmPAG^Tac2^ neurons attenuates aggressive behaviors in mice.

Similarly, we performed chemogenetic silencing of dmPAG^Tac2^ neurons. First, AAV with Cre-dependent inhibitory DREADDs vector (hM4Di-mCherry) was injected into the dmPAG of *Tac2*-Cre mice (Figure 4H-I, referred to as *Tac2*^hM4Di^). *Tac2*-Cre mice injected with a Cre-dependent AAV encoding mCherry alone served as the control group (referred to as *Tac2*^mCherry^). Baseline aggression levels were established by administering normal saline to *Tac2*-Cre mice and subjecting them to the RI test after a 30-minute wait. (Figure 4J). Forty-eight hours later, these mice received an intraperitoneal injection of CNO (1 mg/kg) and were re-tested in the RI assay after a 30-minute wait. As expected, saline administration did not alter fighting behaviors in all mice (Figure 4K-N). However, CNO administration significantly suppressed fighting behaviors in *Tac2*^hM4Di^ mice compared to their baseline test (Figure 4K-N). Inhibition of dmPAG^Tac2^ neurons also significantly reduced the percentage of mice displaying fighting behaviors (Figure S6C).

Of note, we selected a relatively low CNO dose (1 mg/kg) for chemogenetic inhibition based on previous reports suggesting that higher CNO doses (i.e., 5 mg/kg) can elicit non-specific responses in the absence of DREADD receptor expression 45. Consistent with this, we observe no significant effects on locomotor behaviors at the 1 mg/kg dose following dmPAG^Tac2^ neuron inhibition (Figure S7A-D). In the EPM test, CNO administration led to an increased time spent in the open arms, while other anxiety-like measures remained unchanged (Figure S7E-H). In summary, these results suggest that both ablation and inhibition of dmPAG^Tac2^ neurons reduce aggressive behaviors in mice.

### Activation of dmPAG^Tac2^ neurons elicits fighting behaviors

**Figure 5:**
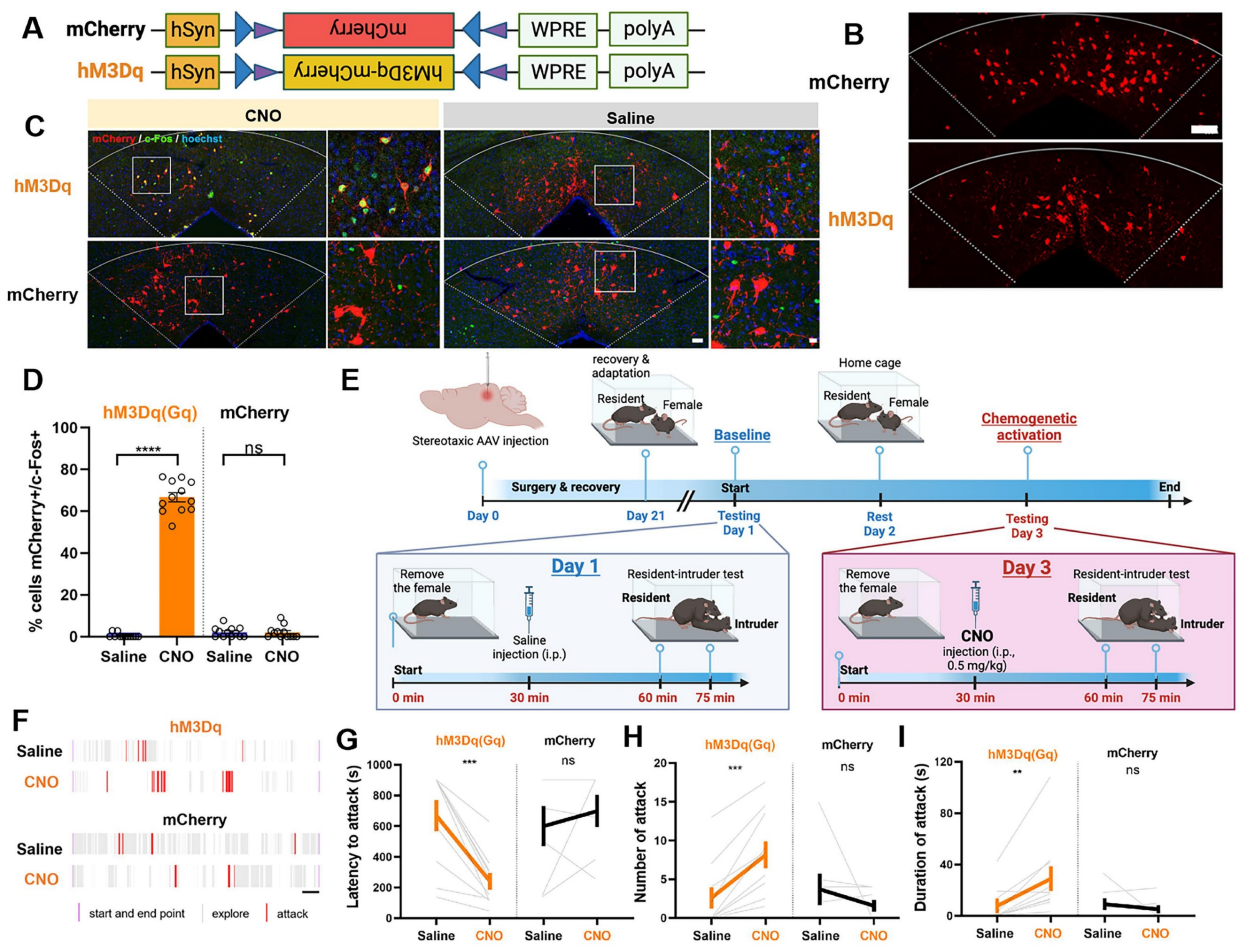
** Chemogenetic activation of dmPAG^Tac2^ neurons elicits fighting behaviors. (A)** A Cre-dependent hM3Dq vector or its control vector to be expressed in dmPAG^Tac2^ neurons. **(B)** Representative fluorescent images of hM3Dq-mCherry (bottom) or mCherry (upper) in dmPAG^Tac2^ neurons. Scale bar, 100 μm. **(C)** Activation of dmPAG^Tac2^ neurons in *Tac2*^hM3Dq^ and control *Tac2*^mCherry^ mice following intraperitoneal administration of CNO and normal saline, respectively (red: mCherry; green: c-Fos; blue: Hoechst). Scale bars, 50 μm (left) and 10 μm (enlarged right panel), respectively. **(D)** Number of mCherry^+^ cells in c-Fos^+^ neurons in *Tac2*-Cre mice receiving CNO injection (0.5 mg/kg, i.p.). N=3 mice, with 4 brain sections from each mouse. **(E)** Strategy for the RI test in *Tac2*-Cre mice receiving DREADDs vectors. **(F)** Raster plots showing mouse attack (red) or exploring behavior (gray) during the RI test. Time scale, 1 minute.** (G-I)** Analysis of latency to attack **(G)**, number of attack **(H)**, and duration of attack **(I)**. N = 10 in *Tac2*^hM3Dq^ group and N = 7 in *Tac2*^mCherry^ group. Data represent mean ± S.E.M. Significance was calculated by means of paired *t*-test. **P < 0.01, ***P < 0.001, ****P < 0.0001.

Having established the necessity of dmPAG^Tac2^ neurons in fighting behaviors through either ablation or inhibition, we proceeded with chemogenetic activation experiments to examine the sufficiency of these neurons in eliciting aggression. A neuron-specific, Cre-dependent AAV encoding hM3Dq was injected into the dmPAG of *Tac2*-Cre mice (referred to as *Tac2*^hM3Dq^, Figure 5A-B). *Tac2*-Cre mice injected with AAV-mCherry served as the control group (referred to as *Tac2*^mCherry^, Figure 5A-B). To confirm the efficacy and specificity of chemogenetic activation, CNO at the dose of 0.5 mg/kg was administered to induce c-Fos induction in Tac2^+^ cells (Figure 5C). Results showed that c-Fos induction was specific to hM3Dq-expressing neurons and was not observed in mCherry-expressing control neurons (Figure 5C-D).

Next, we examined the effects of chemogenetic activation of dmPAG^Tac2^ neurons on fighting behaviors. Baseline aggression levels were established by administering normal saline to *Tac2*-Cre mice, followed by the RI test after a 30-minute wait. Forty-eight hours later, mice received an intraperitoneal injection of CNO (0.5 mg/kg) and were re-tested in the RI assay after a 30-minute wait (Figure 5E). Following saline administration, *Tac2*^mCherry^ mice exhibited no changes in fighting behaviors, consistent with the results observed in chemogenetic inhibition of *Tac2*^mCherry^ mice (Figure 4L-N). However, after CNO administration, *Tac2*^hM3Dq^ mice exhibited significantly elevated aggression levels towards the intruder (Figure 5F-I). We also found that activation of dmPAG^Tac2^ neurons resulted in a threefold increase in the percentage of mice displaying fighting behaviors (Figure S6D). In addition, we assessed the potential effects of CNO administration on other behaviors. No significant changes were observed in locomotor activity (Figure S8A-D) or anxiety-like behaviors (Figure S8E-H) following chemogenetic activation of dmPAG^Tac2^ neurons. Finally, to determine whether chemogenetic manipulation of dmPAG^Tac2^ neurons affects specific aspects of fighting such as chasing, wrestling, and biting, we performed a detailed behavioral analysis. Our analysis showed that inhibition of dmPAG^Tac2^ neurons led to a marked reduction in biting, wrestling, and chasing behaviors (Figure S9A-I). Conversely, activation of these neurons significantly increased biting and wrestling behaviors, with minimal or no effects on chasing behaviors (Figure S10A-I). In summary, activation of dmPAG^Tac2^ neurons specifically elicits fighting behaviors and increases aggression in male mice without affecting other tested behaviors.

### Fighting behaviors alters transcriptional profiles in dmPAG^Tac2^ neurons

**Figure 6:**
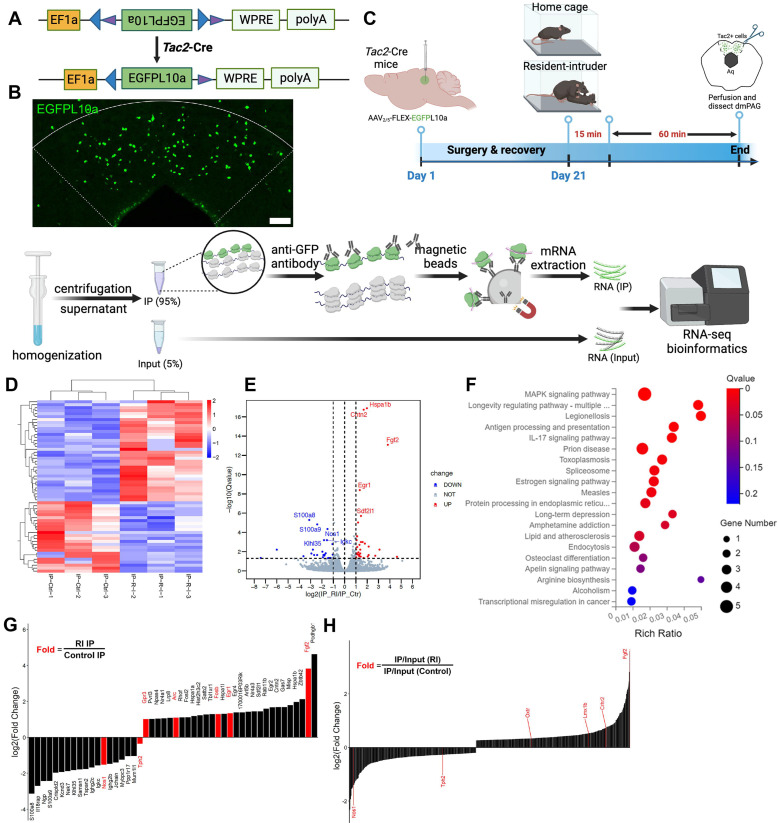
** TRAP-seq reveals transcriptional alterations following fighting behaviors. (A)** A Cre-dependent TRAP (AAV-FLEX-EGFPL10a) vector to be expressed in *Tac2*-Cre mice. **(B)** Representative fluorescent images of EGFPL10a protein (green) in dmPAG. Scale bar, 100 μm. **(C)** Strategy for TRAP-seq in *Tac2*-Cre mice injected with AAV-FLEX-EGFPL10a in dmPAG and subjected to the RI assay or maintained at home cage (control). **(D)** Heatmap showing DEGs of the IP samples. |Log_2_FC|>1, adjusted q value < 0.05. **(E)** Volcano plot showing DEGs of the IP samples. **(F)** KEGG enrichment analysis showing top 20 most enriched pathways affected by RI when compared to the control. **(G)** Fold-change analysis showing log_2_(FC) value of RI-IP to Control-IP samples. Genes labeled in red are immediate-early genes (*Gpr3*, *Arc*, *Fosb*, *Egr1*, *Fgf2*) or genes related to the 5-HT system. **(H)** A secondary analysis of enrichment score, based on normalizing IR/Input (RI) to IR/Input (Control). Note that *Nos1*, *Tph2*, *Oxtr*, *Lmx1b*, *Crhr2*, and *Fgf2* (labeled in red on the left) were listed among the most enriched genes that were related to the 5-HT system.

The above data indicate that dmPAG^Tac2^ neurons play a key role in fighting behaviors, as measured by the RI assay. To investigate the molecular changes in dmPAG^Tac2^ neurons that occur in response to fighting behaviors, we performed the TRAP profiling. A Cre-dependent AAV vector encoding eGFP-tagged ribosome protein L10α was injected into the dmPAG of *Tac2*-cre mice (Figure 6A-B). These mice were either subjected to the RI assay or maintained as home-cage controls (Figure 6C, see *Methods*). Sixty minutes after the RI assay, brain tissues containing the dmPAG were collected. Ribosome-associated mRNAs specific to dmPAG^Tac2^ neurons were purified and RNA-sequencing (RNA-seq) was performed. By comparing gene expression between the two groups of immunoprecipitated (IP) samples (RI vs. Control), we identified 58 differentially expressed genes (DEGs) associated with fighting, including 34 upregulated and 23 downregulated DEGs (Figure 6D-E). Kyoto Encyclopedia of Genes and Genomes (KEGG) analysis revealed that fighting behaviors primarily impacted inflammation, immunity and infection-related pathways (Figure 6F). Of note, the estrogen signaling pathway was identified, aligning with previous studies showing that estrogen signaling in the VMH can regulate inter-male aggression 11, supporting the notion that estrogen modulates aggression behaviors 22, 46, 47.

To identify novel targets related to fighting behaviors, we performed enrichment analysis 48 by calculating fold enrichment (RI-IP/Ctr-IP) and highlighting the top candidate genes (Figure 6G-H). Among the most significantly enriched genes, several well-established immediate-early genes, including *Arc*, *Egr1*, *Fgf2*, and *Fosb*, were upregulated. This finding indicates that dmPAG^Tac2^ neurons were indeed activated by fighting behaviors, validating the TRAP-seq approach in capturing relevant molecular events.

Interestingly, several downregulated genes were also identified, notably *Tph2*, which encodes tryptophan hydroxylase 2, a rate-limiting enzyme primarily expressed in serotonergic neurons. This finding aligns with previous evidence implicating the serotonergic system in aggression at both ligand and receptor levels 49-52, suggesting a potential link between reduced 5-HT synthesis and increased aggression in mice. Moreover, a secondary comparative analysis between the aggression group (IP versus Input ratio) and the control homecage group revealed additional aggression-associated genes (Figure 6H), many of which also relate to the 5-HT system. Taken together, these findings suggest that the 5-HT pathway may play a critical role in dmPAG^Tac2^ neuron's involvement in aggressive behavior. Thus, we decided to choose the 5-HT system for our subsequent pharmacological validation experiment.

### Pharmacological manipulation of dmPAG^Tac2^ neurons in fighting behaviors

**Figure 7:**
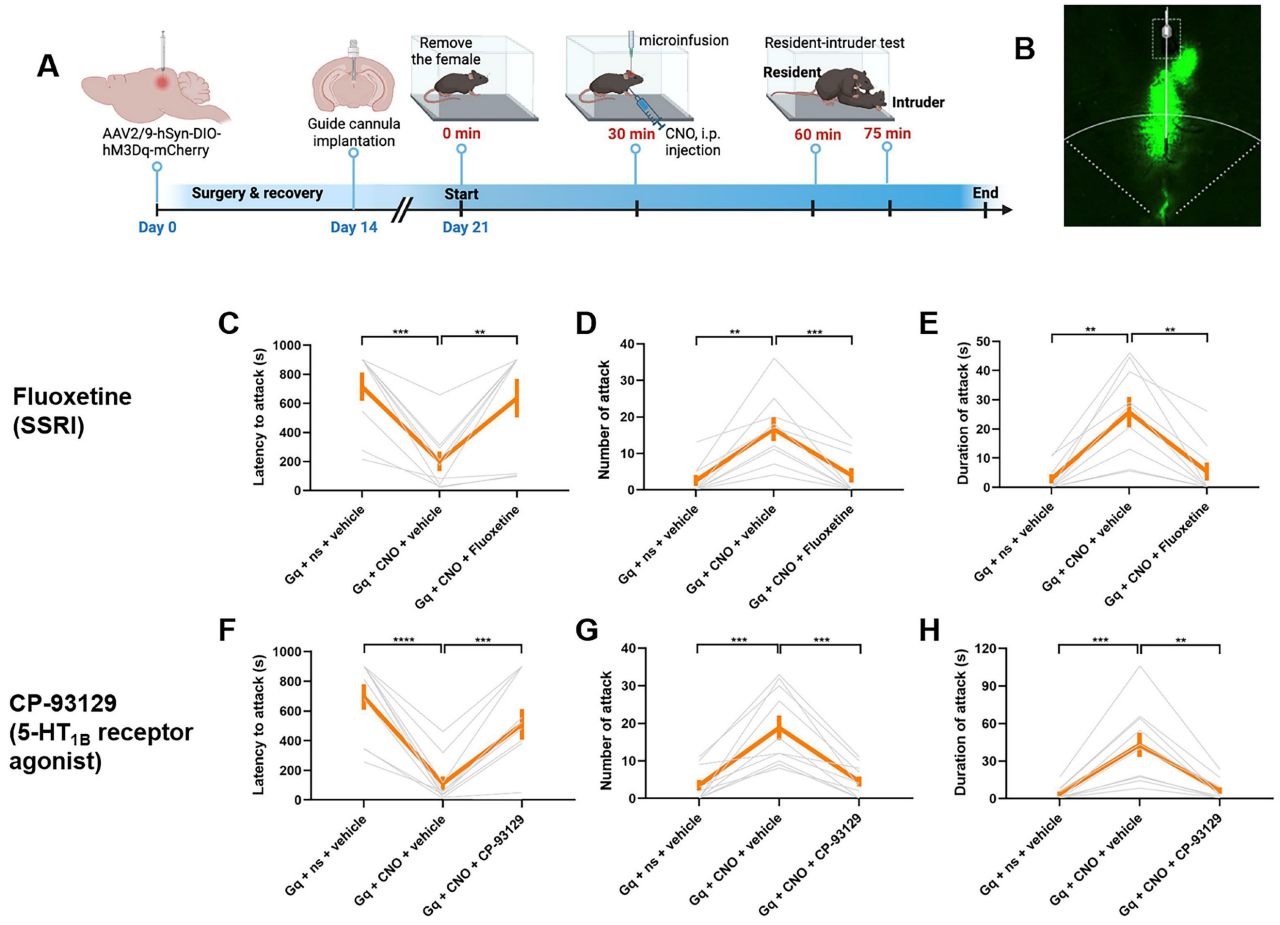
** Pharmacological validation of molecular targets identified by TRAP-seq. (A)** Strategy for pharmacological manipulation of the 5-HT system with fluoxetine or CP-93129 to alter fighting behaviors. **(B)** Representative fluorescent image showing cannula position targeting dmPAG region in *Tac2*-Cre mice. **(C-E)** Analysis of latency to attack **(C)** number of attack **(D)** and duration of attack **(E)** in *Tac2*^hM3dq^ mice injected with fluoxetine/vehicle and CNO/saline in the dmPAG. N = 9 mice/group. **(F-H)** Analysis of latency to attack **(F)** number of attack **(G)** and duration of attack **(H)** in *Tac2*^hM3dq^ mice injected with CP-93129/vehicle and CNO/saline in the dmPAG. N = 10 mice/group. Data represent mean ± SEM. Significance was calculated by means of paired *t*-test. *P < 0.05, **P < 0.01, ***P < 0.001, ****P < 0.0001.

To validate the potential molecular pathways in dmPAG^Tac2^ neuron altered by fighting behaviors, we targeted the 5-HT system as identified by TRAP-seq. We hypothesized that inhibiting 5-HT reuptake, thereby increasing extracellular 5-HT levels in the synaptic cleft, could block the behavioral responses induced by chemogenetic activation of dmPAG^Tac2^ neurons, as previously demonstrated 53, 54. To test this, we administered fluoxetine, a selective 5-HT reuptake inhibitor (SSRI), intracranially into the dmPAG prior to chemogenetic activation (Figure 7A-B). As expected, CNO administration in *Tac2*^hM3Dq^ mice effectively elicited fighting behaviors, while intra-dmPAG delivery of fluoxetine prevented these behaviors (Figure 7C-E). Specifically, fluoxetine delivery resulted in a significantly increased latency to attack (Figure 7C), a decreased number (Figure 7D) and duration of attack (Figure 7E).

Given the critical roles of specific 5-HT receptor subtypes, such as the 5-HT_1B_ receptor, in modulating aggression 55, 56, we next investigated how modulation of the 5-HT_1B_ receptor using specific agonist and antagonist can affect fighting behaviors. First, we intracranially administered CP-93129, a selective 5-HT_1B_ receptor agonist, directly into the dmPAG before chemogenetic activation. As expected, CNO administration in Tac2^hM3Dq^ mice effectively elicited fighting behaviors, while administration of CP-93129 successfully prevented these effects, demonstrated by a longer latency to attack (Figure 7F), along with fewer attack (Figure 7G) and shorter attack duration (Figure 7H). Conversely, we tested the effect of 5-HT inhibition on fighting behaviors by delivering SB-224289, a selective 5-HT_1B_ receptor antagonist, into the dmPAG prior to chemogenetic inhibition. CNO administration in Tac2^hM4Di^ mice suppressed fighting behaviors. However, intra-dmPAG administration of SB-224289 counteracted this effect, leading to more rapid initiation of attack (Figure S11A), as well as an increased number (Figure S11B) and duration of attack (Figure S11C). Additionally, we analyzed the TRAP-seq data focusing on 5-HT system-associated genes, including those encoding 5-HT receptors (Figure S11D-G). The analysis reveals that RI exposure downregulated the synthesis of 5-HT (Figure S11D-E) and upregulated the expression of several 5-HT receptor genes (Figure S11F-G), supporting a role for the 5-HT system in dmPAG^Tac2^ neuron-mediated fighting behaviors. Together, these results suggest that modulating 5-HT or 5-HT_1B_ receptor in dmPAG neurons can influence fighting behaviors elicited by activation or inhibition of dmPAG^Tac2^ neurons, thus highlighting a potential role of the 5-HT system in dmPAG^Tac2^-mediated aggression.

## Discussion

### dmPAG^Tac2^ neurons exhibit distinct expression pattern

Tac2^+^ neurons display a unique distribution pattern in the mouse brain. Under basal level, *Tac2* genes are expressed in a wide variety of brain regions associated with mood and social behaviors, including the bed nuclei of the stria terminalis (BNST), medial habenular (MHb), central amygdala (CeA) 10. In pathological states, such as prolonged social isolation, Tac2 expression can be strongly induced in a dissociable and region-specific manner 10. This induction pattern of Tac2 peptide and distributed expression of Tac2^+^ neurons suggest that the regulatory roles of the Tac2/NkB signaling pathway depend on the anatomical location and neural connection of Tac2^+^ neurons. For instance, *Tac2*-expressing neurons in the dorsomedial shell of the nucleus accumbens have been characterized as a subtype of D1 medium spiny neurons that play a regulatory role in cocaine addiction 57. In the MHb, Tac2^+^ neurons regulate mood-related behaviors 43, 58 but in the CeA, Tac2^+^ neurons are involved in the consolidation of fear memory 9, 59. Recent research also suggests that Tac2^+^ neurons in the BNST and CeA regulate emotional valence by sending inhibitory inputs to melanin-concentrating hormone (MCH)-expressing neurons in the LHA 60. These findings highlight the region-specific roles of Tac2^+^ neurons in modulating diverse behavioral functions.

While Tac2 expression occurs across various brain regions, its specific functions may differ by location. Our study focused on Tac2^+^ neurons located in the PAG, where we observed these neurons were primarily located in the dorsomedial part (dmPAG), a region associated with defensive responses, such as panic, fight-flight, and freezing behaviors 1, 7. Previous studies indicated an anatomic segregation of defensive behaviors in the dorsal PAG columns 61, with the dmPAG acting as a relay station for aversive information to other brain regions 62. Here, we uncovered dmPAG^Tac2^ neurons as a molecularly distinct cell type critically involved in intermale aggression. Notably, Tac2^+^ cells are also present in other regions, such as the MHb, although we did not assess their potential role in aggression. Future studies could investigate whether Tac2^+^ cells in the MHb contribute to aggression-related behaviors or other social interactions.

Building on the unique region-specific roles of Tac2^+^ neurons, we further examined the expression pattern and behavioral relevance of Tac2^+^ neurons in the dmPAG, a region critically associated with defensive and aggression-related behaviors. While our study primarily examines the role of dmPAG^Tac2^ neurons in aggression, it worth noting that revealing the broader neural circuits that modulate their activity is crucial for a deep understanding of their function. Previous studies suggest that the dmPAG receives substantial inputs from hypothalamic nuclei, particularly the ventromedial hypothalamus (VMH), posterior hypothalamic nucleus (PH), and medial preoptic area (mPOA), but to the lesser degree from regions such as the posterior prelimbic cortex (PrL) and central amygdala (CeA), many of which are implicated in aggression 2, 8, 63, 64. On the other hands, the dmPAG are reported to send inputs to downstream targets, including the nucleus raphe magnus (NRM), ventrolateral medulla (VLM), median raphe nucleus (MnR), dorsal raphe nucleus (DR), and mPOA, which are involved in motor and emotional regulation 65, 66. These regions can integrate sensory and emotional signals to modulate the initiation of aggressive behavior. Thus, understanding the connections between Tac2^+^ neurons in the dmPAG and upstream or downstream structures could help clarify the mechanisms that mediate the transition from sensory perception of threats to aggression. Future studies using monosynaptic rabies viral tracing, optogenetics, or chemogenetics will be critical to dissect these circuits and test how manipulation of upstream or downstream pathways can modulate dmPAG^Tac2^ activity and behavioral responses such as aggression.

### dmPAG^Tac2^ neurons are recruited during intermale aggression

Having confirming the specific localization of Tac2^+^ neurons in the dmPAG, we hypothesized that these neurons play a role in aggression. Using a RI paradigm, we found that fighting behaviors selectively activated dmPAG^Tac2^ neurons, as shown by c-Fos induction and time-locked Ca^2+^ transient detected in fiber photometry experiments (**Figure 3**). These findings suggest that dmPAG^Tac2^ neurons are recruited during aggression encounters.

Previous studies have demonstrated a sex-specific effect of Tac2^+^ neurons in the CeA 8, 22, 59. Although we focused on dmPAG^Tac2^ neurons in intermale aggression, future research should investigate their role in other types of aggressions, such as maternal and predatory aggression. The PAG is also known to regulate predatory aggression 67, with the lPAG involved in sensory processing 68 and vlPAG glutamatergic neurons in conditioned freezing responses 69. Whether dmPAG^Tac2^ neurons participate in predatory aggression remains unknown and warrants future study.

### Bidirectional manipulation of dmPAG^Tac2^ neuronal activity alters fighting behaviors

Our experiments with targeted inhibition and genetic ablation of dmPAG^Tac2^ neurons showed that these neurons are necessary for fighting behaviors. Inhibiting dmPAG^Tac2^ activity increased the latency to attack and significantly reduced both the number and duration of attack in the RI assay (**Figure 4**), indicating suppressed aggression. On the other hand, chemogenetic activation of dmPAG^Tac2^ neuronal activity shortened attack latency and markedly increased both number and duration of attack (**Figure 5**), demonstrating that dmPAG^Tac2^ neuron activation is sufficient to induce aggression. This bidirectional evidence underscores the critical role of dmPAG^Tac2^ neurons in modulating fighting behaviors in male mice. In chemogenetic manipulation, we employed a before-and-after study design, as used in previous studies 11, 23, 26 to maximize the assessment of intervention effects on individual animals, thereby providing robust baseline and post-intervention comparisons 70.

### Role of the 5-HT system in dmPAG^Tac2^ neurons during aggression

The relationship between the 5-HT system and aggression is well-documented. Prior studies have shown that genetic alterations in the 5-HT system can affect aggressive behaviors, such as heightened aggression in male mice with monoamine oxidase A mutations and altered brain 5-HT levels 71, 72, or intense aggression in mice lacking the 5-HT_1B_ receptors 73. Our study suggests potential involvement of the 5-HT system in dmPAG^Tac2^ neurons-mediated aggression. However, our evidence primarily relies on pharmacological interventions with agents targeting the 5-HT system and correlative transcriptomic data, thus, further studies are needed to confirm the specificity of this pathway.

Our TRAP-seq analysis revealed changes in transcripts related to 5-HT signaling in dmPAG^Tac2^ neurons following RI exposure. Although pharmacological manipulation of 5-HT signaling pathway could modulate aggression elicited by dmPAG^Tac2^ activation or inhibition, which align with previous reports demonstrating the involvement of 5-HT system in aggression 71-73, one should be noted that, these findings are preliminary. They suggest a possible link between 5-HT pathways and dmPAG^Tac2^ neurons but require further validation, whereas additional studies are needed to elucidate the precise mechanisms by which 5-HT signaling intersects with the Tac2/NkB pathway in the dmPAG.

### Limitations

In clinical assessment, aggression is often categorized into subtypes, such as impulsive or premeditated aggression 74. In animal studies, aggressive and defensive behaviors are typically classified more broadly, encompassing play fighting, offensive aggression, defensive aggression, predatory aggression 24,75. A limitation of our study is that we primarily focused on intermale aggression, without examing other types of aggression, such as female, maternal, or predatory aggression. Recent studies have highlighted a sex-dependent role of Tac2^+^ neurons in fear memory regulation 59, suggesting the importance of investigating sexual dimorphism in future research.

### Potential treatment for humans with exaggerated aggression

Uncontrolled aggression poses significant risks to society, including physical harm and chronic stress, both of which can negatively impact mental health 76. In severe cases, such aggression can escalate to serious incidents like homicide or mass violence 77. Developing effective strategies to manage aggression is thus crucial. Our study identifies a molecularly distinct neuronal cell type in the midbrain that regulates intermale aggression in mice. While our findings suggest that this cell type could be a potential target for pharmacological intervention, it is important to note that, we have yet to identify a specific genetic target. Additionally, we acknowledge that the resident-intruder paradigm is an animal model and may not fully capture the complexity of human aggression. These limitations underscore the need for further research to explore the translational potential of our findings.

## Supplementary Material

Supplementary figures.

## Abbreviations

5-HT: 5-hydroxytryptamine
7-NI: 7-Nitroindazole
AAV: adeno-associated virus
ACC: anterior cingulate cortex
AHN: anterior hypothalamic nucleus
ARH: arcuate hypothalamic nucleus
BLA: basolateral amygdalar nucleus
BNST: bed nuclei of the stria terminalis
CeA: central amygdalar nucleus
CNO: clozapine N-oxide
CUN: cuneiform nucleus
DEPC: diethypyrocarbonate
dIPAG: dorsolateral periaqueductal gray
DMH: dorsomedial nucleus of the hypothalamus
dmPAG: dorsomedial periaqueductal gray
DMSO: dimethyl sulfoxide
DR: dorsal nucleus raphe
DTA: diphtheria toxin
EGFP: enhanced green fluorescent protein
EPM: elevated plus maze
GABA: γ-aminobutyric acid
HPF: hippocampal formation
LHA: lateral hypothalamic area
LPAG: lateral periaqueductal gray
MeA: medial amygdalar nucleus
MHb: medial habenula
MM: medial mammillary nucleus
MPN: medial preoptic nucleus
MPO: medial preoptic area
MRN: midbrain reticular nucleus
NAc: nucleus accumbens
NK: neurokinin
nNOS: neuronal nitric oxide synthase
OB: olfactory bulb
OCT: optimal cutting temperature compound
OFT: open field test
PAG: periaqueductal gray
PBS: phosphate buffer saline
PVT: paraventricular nucleus of the thalamus
SSRI: selective serotonin reuptake inhibitor
Tac: tachykinin
TPH2: tryptophan hydroxylase2
TRAP: translating ribosome affinity purification
VIPAG: ventrolateral periaqueductal gray
VMH: ventromedial hypothalamic nucleus
