# Supplementary material for: A molecularly distinct cell type in the midbrain regulates intermale aggression behaviors in mice
Source: Theranostics. 2025 Jan 1;15(2):707–25. doi: 10.7150/thno.101658 (PMC11671387; doi:10.7150/thno.101658)
Supplement: Supplementary file 1 — Supplementary figures. [file thnov15p0707s1.pdf]

Supplementary Figure Legends

Figure S1. **PAG *Tac2*-expressing neurons are mainly located in the dmPAG** (related to Figure 1).

(A) Representative fluorescent images showing absence of tdTomato-labeled Tac2$^+$ cells in control mice (*Tac2*-Cre, Ai9, or WT C57BL/6 mice). Scale bars, 500 μm. **(B)** *Tac2* mRNA expression pattern in ABA ISH database (left), with an enlarged view (right) from the left panel. **(C)** Distribution pattern of Tac2$^+$ cells in PAG subregions examined fluorescence ISH. Scale bars, 50 μm. **(D)** Pie chart showing numbers of $Tac2^{tdTomato}$ cells in PAG subregions. N = 3 mice. Data were expressed as mean ± S.E.M.

Figure S2. **Mapping of c-Fos expression in WT mice subjected to the RI assay** (related to Figure 2)

(A) Brain areas with high-level c-Fos expression in WT mice subjected to the RI assay. Scale bar, 1,000 μm. **(B)** Representative fluorescent image showing multiple hypothalamic subnuclei were activated by the RI assay, including the PVT, SBPV, PVZ, AHN, LHA, BLA, DMH, VMH, MRN, and PAG. Scale bar, 100 μm. **(C)** Quantification of number of Fos$^+$ cells in selected brain areas in WT mice subjected to the RI assay. Data are presented as total number of Fos$^+$ cells in five brain sections separated by 200 μm (N = 3 mice per condition). Data were expressed as mean ± S.E.M. *Abbreviations*: PVT, Paraventricular nucleus of the thalamus; SBPV, Subparaventricular zone hypothalamus; PVZ, Periventricular zone; AHN, Anterior hypothalamic nucleus; LHA, Lateral hypothalamic area; BLA, Basolateral amygdala nucleus, DMH, Dorsomedial nucleus of the hypothalamus; VMH, Ventromedial hypothalamic nucleus; MRN, Midbrain reticular nucleus; PAG, Periaqueductal gray.

Figure S3. **Using a Cre-out strategy to test the role of dmPAG^{Tac2} negative neurons in aggression** (related to Figure 3)

(A) Schematic showing a Cre-out strategy. **(B-D)** Ca^{2+} activity time-locked to each attack episode. The panels showing **(B)** Distribution of attack episode, **(C)** Heatmap of GCaMP6m, and **(D)** $\Delta F/F$%. **(E)** Analysis of peak $\Delta F/F$ before (Pre) and after (Post) each attack episode. Data represent mean ± S.E.M. Significance was calculated by means of paired *t*-test. **P* < 0.05. **(F)** Analysis of peak $\Delta F/F$ in dmPAG^{Tac2} neurons (N = 10 trails, in Figure 3H) versus dmPAG^{Tac2} negative neurons (N = 46 trails) during each attack episode. Data represent mean ± S.E.M. unpaired *t*-test, *****P* < 0.0001.

Figure S4. **Tac2-expressing neurons in dmPAG respond to aggression** (related to Figure 3)

(A-F) Ca^{2+} activity time-locked to mice encounter **(A-B)**, male-female encounter **(C-D)**, and male-female interaction **(E-F)**. **(A)** Heatmap of GCaMP6m $\Delta F/F$ signals (top) and $\Delta F/F$% (bottom). **(B)** Analysis of peak $\Delta F/F$ before (Pre) and after (Post) each male-male encounter episode. The other panels present the same manner but with different behavioral events, including male-female encounter **(C-D)** and male-female interaction **(E-F)**. **(G-H)** Ca^{2+} activity time-locked to motor activity in the rotarod test. **(G)** Heatmaps of GCaMP6m $\Delta F/F$ signals (top) and $\Delta F/F$% (bottom). (H) Analysis of peak $\Delta F/F$ before (Pre) and after (Post) the start of rotarod test. **(I-P)** Ca^{2+} activity time-locked to object exploration **(I-J)**, fox odor sniffing **(K-L)**, saline odor sniffing **(M-N)**, and pain stimulation **(O-P)**. **(I)** Distribution of object exploration (top), heatmaps of GCaMP6m $\Delta F/F$ signals (middle) and $\Delta F/F$% (bottom). **(J)** Analysis of peak $\Delta F/F$ before (Pre) and after (Post) each object exploration episode. The other panels present the same manner but with different behavioral events, including fox odor sniffing **(K-L)**, saline odor sniffing **(M-N)**, and pain stimulation **(O-P)**. Data represent mean ± S.E.M. Significance was calculated by means of paired *t*-test.

Figure S5. Behavioral phenotypes (locomotion, anxiety, social behaviors) in *Tac2*-Cre mice (related to Figure 4).

(A) Schematic of the open-field test. **(B-E)** Locomotor performance of WT and *Tac2*-Cre mice on **(B)** total distance, **(C)** average speed, **(D)** time spent in central and peripheral zones, and **(E)** entries into the central and peripheral zones. **(F)** Schematic of the elevated-plus maze test. **(G-J)** Anxiety-like behaviors of WT and *Tac2*-Cre mice on **(G)** numbers of entries in closed arm, **(H)** time spent in closed arm, **(I)** numbers of entries in open arm, **(J)** time spent in open arm. **(K)** Schematic of the social interaction test (sociability session). **(L-M)** Social preference of WT and *Tac2*-Cre mice on **(L)** interaction time and **(M)** social preference index. **(N)** Schematic of the social interaction test (social novelty session). **(O-P)** Social preference of WT and *Tac2*-Cre mice on **(O)** interaction time and **(P)** social preference index. **(Q)** Schematic of the RI assay. **(R-T)** Behavioral performance of WT and *Tac2*-Cre mice on (R) latency to attack, **(S)** number of attack and **(T)** duration of attack. Data represent mean ± S.E.M. N = 24 in WT mice and N = 25 in *Tac2*-Cre mice. Unpaired t-test, *$P < 0.05$, ***$P < 0.001$, ****$P < 0.0001$.

Figure S6. Percentage of *Tac2*-Cre mice exhibit attack behaviors (related to Figure 4-5).

(A) Percent of mice exhibiting attack before and after genetic ablation with DTA or mCherry (control). (**B**) Number (left) and duration (right) of investigation before and after ablation of $dmPAG^{Tac2}$ neurons with DTA in *Tac2*-cre mice. N = 7 mice. Data represent mean ± S.E.M. Significance was calculated by means of paired *t*-test. **(C)** Percent of mice exhibiting attack before and after receiving hM4Di or mCherry (control). N = 10 in $Tac2^{hM3Dq}$ and N = 7 in $Tac2^{mCherry}$ groups. **(D)** Percent of animals exhibiting attack before and after hM3Dq or mCherry (control). N = 10 in $Tac2^{hM4Di}$

and N = 7 in *Tac2*^{mCherry} group. Data represent mean ± S.E.M. Significance was calculated by One-way ANOVA and Tukey's multiple comparisons test,

Figure S7. **Behavioral phenotypes (locomotion and anxiety behaviors) following chemogenetic inhibition** (related to Figure 4).

(A-D) Performance of the open-field test in *Tac2*^{hM4Di} mice receiving saline or CNO (1 mg/kg, *i.p.*). Analysis showing results of total distance **(A)**, average speed **(B)**, time spent in the central or peripheral zones **(C)**, and entries into the central or peripheral zones **(D)**. **(E-H)** Performance of the elevated-plus maze in *Tac2*^{hM4Di} mice receiving saline or CNO (1 mg/kg, *i.p.*). Analysis showing results of entries in the open arm **(E)**, total time spent in the open arm **(F)**, entries in the closed arm **(G)**, and total time spent in the closed arm **(H)**. N = 10 mice. Data represent mean ± S.E.M. Significance was calculated by means of paired *t*-test, $*P < 0.05$.

Figure S8. **Detailed analysis of specific components of fighting behaviors following chemogenetic inhibition experiments** (related to Figure 4).

(**A-C**) Analysis of chasing behaviors, showing the latency to exhibit chasing (**A**), number of chasing (**B**), and duration of chasing (**C**). (**D-F**) Analysis of wrestling behaviors, showing latency to exhibit wrestling (**D**), number of wrestling (**E**), and duration of wrestling (**F**). (**G-I**) Analysis of biting behaviors, showing latency to exhibit biting (**G**), number of biting (**H**), and duration of biting (**I**). N = 10 in *Tac2*^{hM4Di} group and N = 7 in *Tac2*^{mCherry} group. Data represent mean ± S.E.M. Significance was calculated by means of paired *t*-test. $**P < 0.01$, $***P < 0.001$, $****P < 0.0001$.

Figure S9. **Behavioral phenotypes (locomotion and anxiety behaviors) following chemogenetic activation** (related to Figure 5).

(A-D) Performance of the open-field test of *Tac2*^{hM3Dq} mice receiving saline or CNO (0.5 mg/kg, *i.p.*). Analysis showing results of total distance **(A)**, average speed **(B)**, total time in the central or peripheral zones **(C)**, and entries in the central or peripheral zones **(D)**. **(E-H)** Performance of elevated-plus maze of *Tac2*^{hM3Dq} mice receiving saline or CNO (0.5 mg/kg, *i.p.*). Analysis showing results of entries in the open arm **(E)**, total time spent in the open arm **(F)**, entries in the closed arm **(G)**, and total time spent in the closed arm **(H)**. N = 8 mice. Data represent mean ± S.E.M. Significance was calculated by means of paired *t*-test.

Figure S10. **Detailed analysis of specific components of fighting behaviors following chemogenetic activation experiments** (related to Figure 5).

(**A-C**) Analysis of chasing behaviors, showing the latency to exhibit chasing (**A**), number of chasing (**B**), and duration of chasing (**C**). (**D-F**) Analysis of wrestling behaviors, showing latency to exhibit wrestling (**D**), number of wrestling (**E**), and duration of wrestling (**F**). (**G-I**) Analysis of biting behaviors, showing latency to exhibit biting (**G**), number of biting (**H**), and duration of biting (**I**). N = 10 in $Tac2^{\mathrm{hM3Dq}}$ group and N = 7 in $Tac2^{\mathrm{mCherry}}$ group. Data represent mean ± S.E.M. Significance was calculated by means of paired *t*-test. *$P < 0.05$, **$P < 0.01$, ***$P < 0.001$.

Figure S11: **Pharmacological validation of molecular targets identified by TRAP-seq** (related to Figure 7)

(A-C) Analysis of latency to attack **(A)** number of attack **(B)** and duration of attack **(C)** in $Tac2^{\mathrm{hM4di}}$ mice injected with SB-224289/vehicle and CNO/saline in the dmPAG. N = 9 mice/group. **(D)**. Heatmap showing counts of *Tph2* from TRAP-seq raw results. **(E)** Heatmap showing Log_2FC value of *Tph2* transcript counts in individual sample pairs. **(F)** Counts of 5-HTR related genes detected in the IP samples from RI mice and home-cage controls. **(G)** Log_2FC value in the counts of 5-HTR related genes in the IP samples from RI mice normalized to that of the home-cage control mice. Data represent mean ± SEM. Significance was calculated by means of paired *t*-test. *$P < 0.05$, **$P < 0.01$, ***$P < 0.001$.

Figure S1

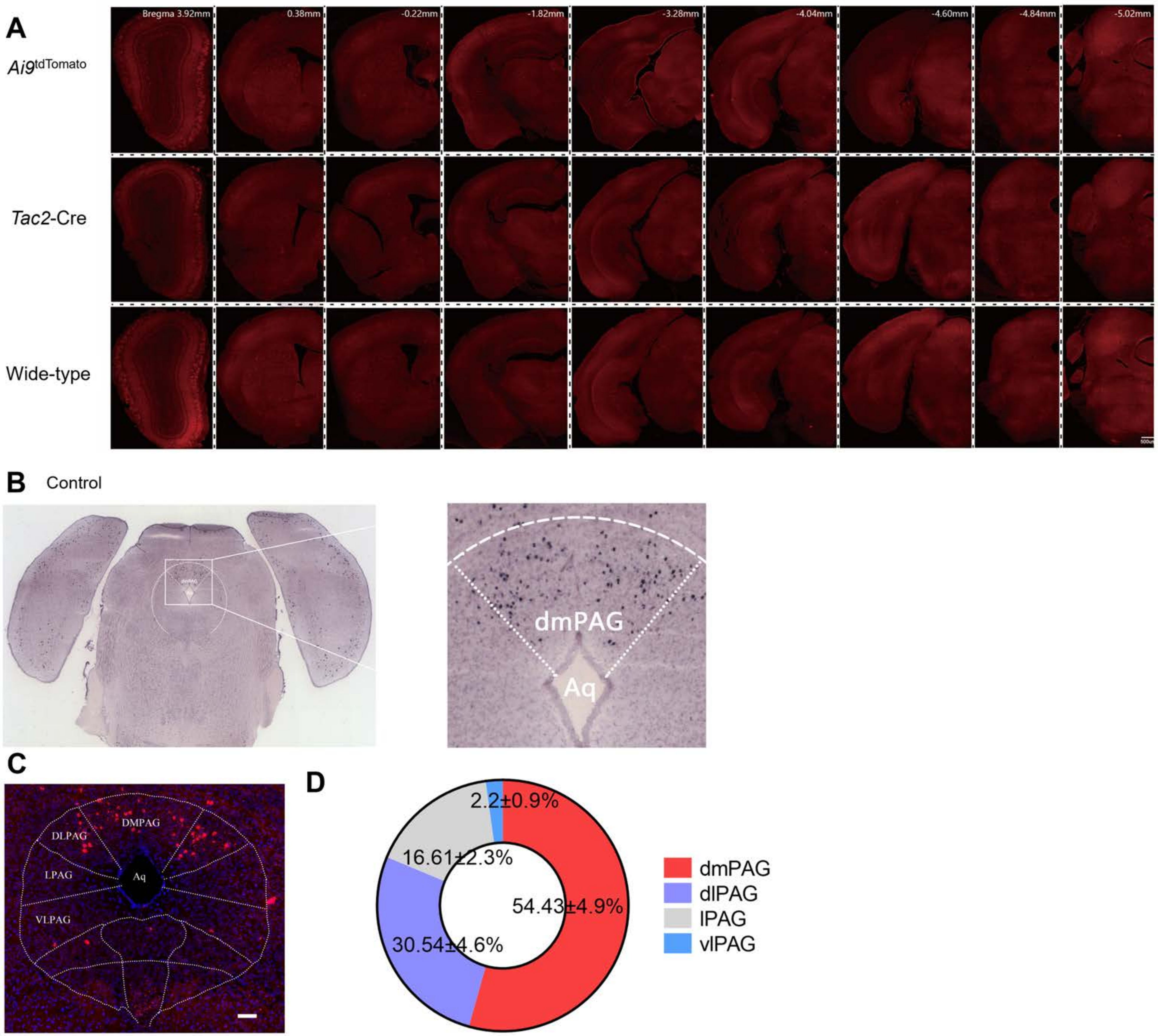

A
Ai9tdTomato
Tac2-Cre
Wide-type
Bregma 3.92mm
0.38mm
-0.22mm
-1.82mm
-3.28mm
-4.04mm
-4.60mm
-4.84mm
-5.02mm
B
Control
dmPAG
Aq
C
DMPAG
DLPAG
LPAG
VLPAG
Aq
D
2.2±0.9%
16.61±2.3%
54.43±4.9%
30.54±4.6%
dmPAG
dlPAG
lPAG
vlPAG

Figure S2

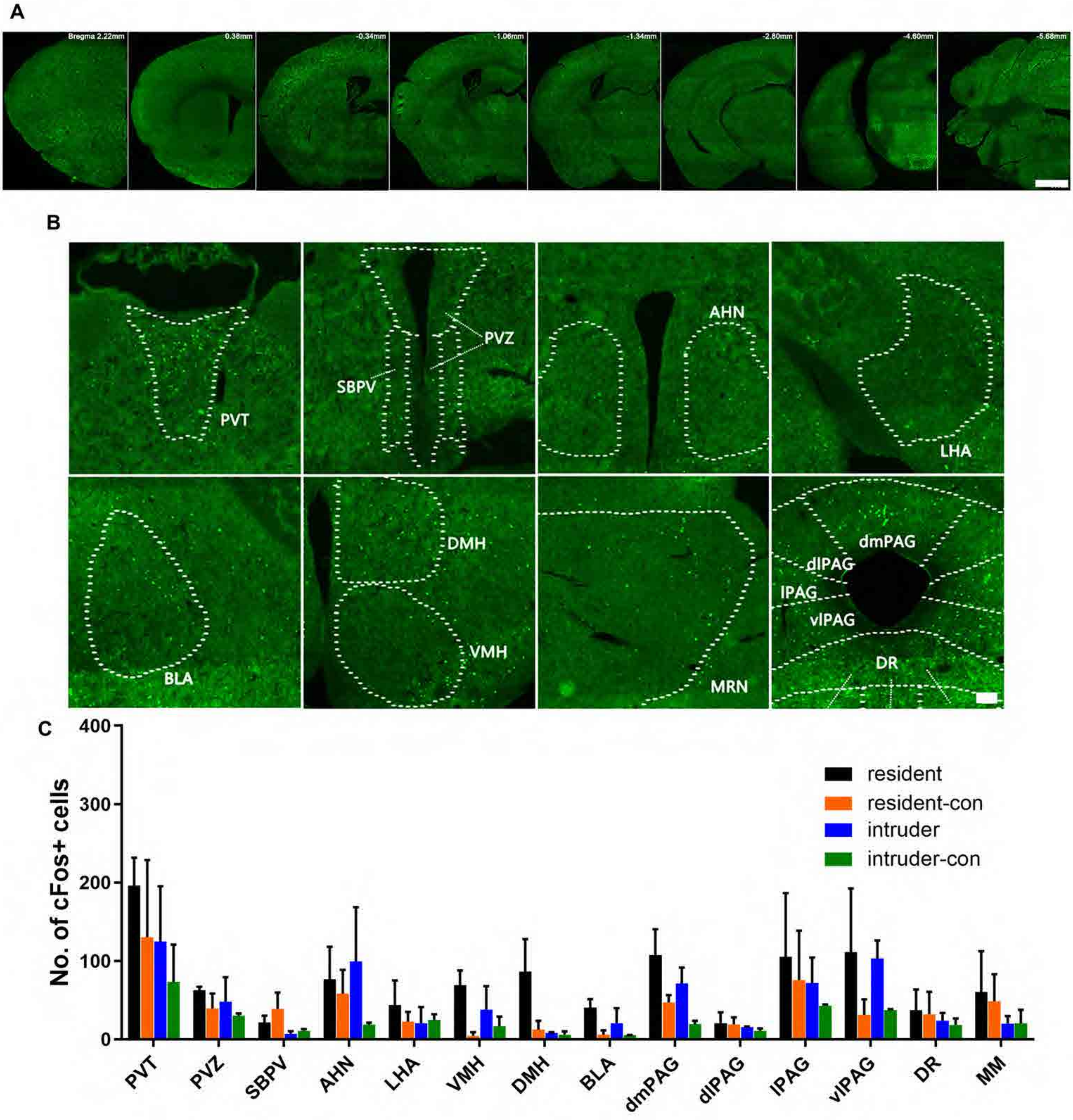
A
Bregma 2.22mm
0.38mm
-0.34mm
-1.06mm
-1.34mm
-2.80mm
-4.60mm
-5.68mm
B
PVT
PVZ
SBPV
AHN
LHA
DMH
VMH
BLA
MRN
dmPAG
dlPAG
lPAG
vlPAG
DR
C
No. of cFos+ cells
0
100
200
300
400
resident
resident-con
intruder
intruder-con
PVT
PVZ
SBPV
AHN
LHA
VMH
DMH
BLA
dmPAG
dlPAG
lPAG
vlPAG
DR
MM

Figure S3

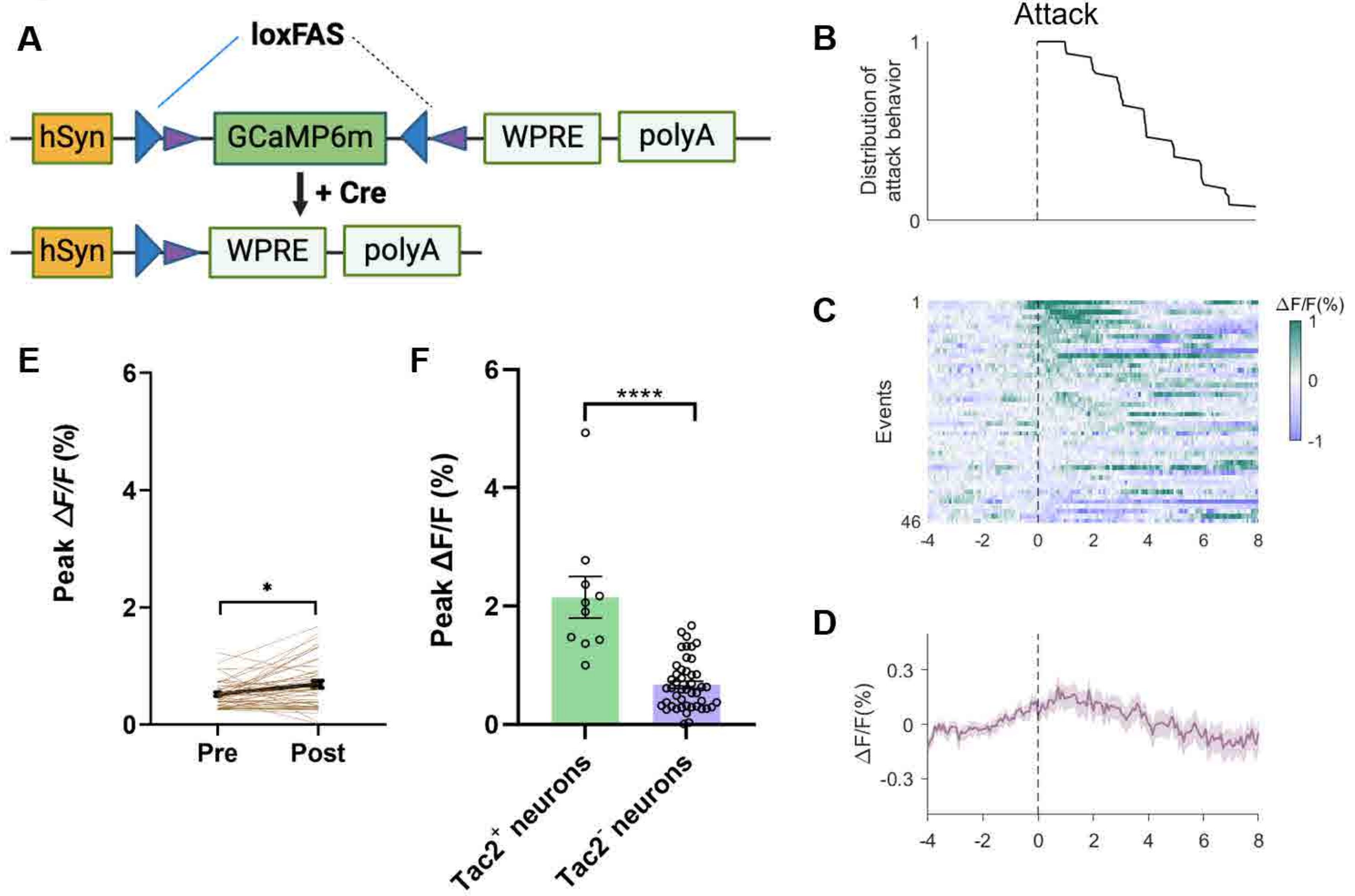

A
loxFAS
hSyn
GCaMP6m
WPRE
polyA
+ Cre
hSyn
WPRE
polyA
B
Attack
Distribution of attack behavior
1
0
C
1
46
Events
ΔF/F(%)
1
0
-1
-4 -2 0 2 4 6 8
D
ΔF/F(%)
0.3
0
-0.3
-4 -2 0 2 4 6 8
E
Peak ΔF/F (%)
6
4
2
0
*
Pre
Post
F
Peak ΔF/F (%)
6
4
2
0
****
Tac2+ neurons
Tac2- neurons

Figure S4

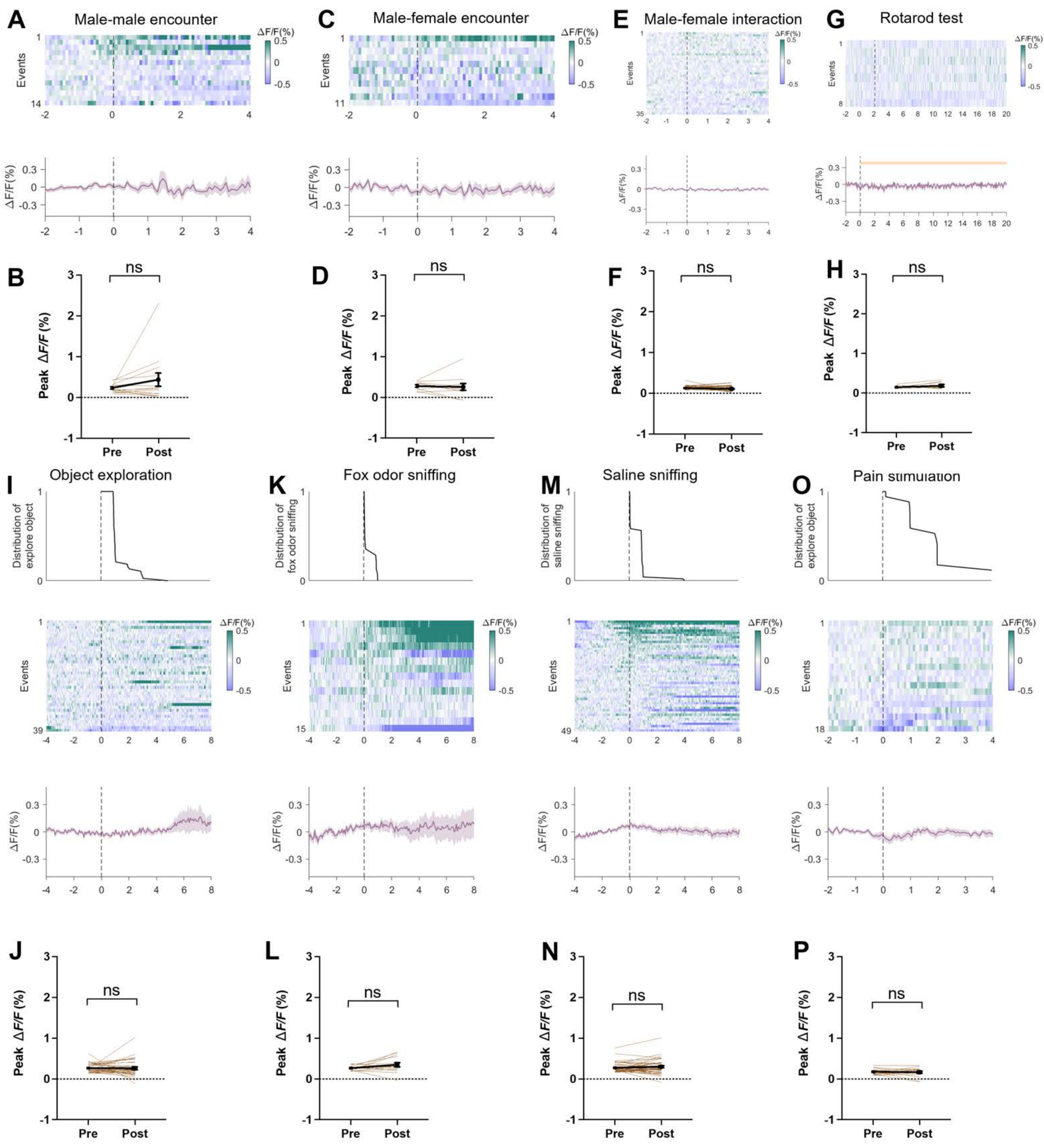

A
Male-male encounter
C
Male-female encounter
E
Male-female interaction
G
Rotarod test
Events
ΔF/F(%)
B
D
F
H
ns
Peak ΔF/F (%)
Pre
Post
I
Object exploration
K
Fox odor sniffing
M
Saline sniffing
O
Pain stimulation
Distribution of explore object
Distribution of fox odor sniffing
Distribution of saline sniffing
J
L
N
P

Figure S5

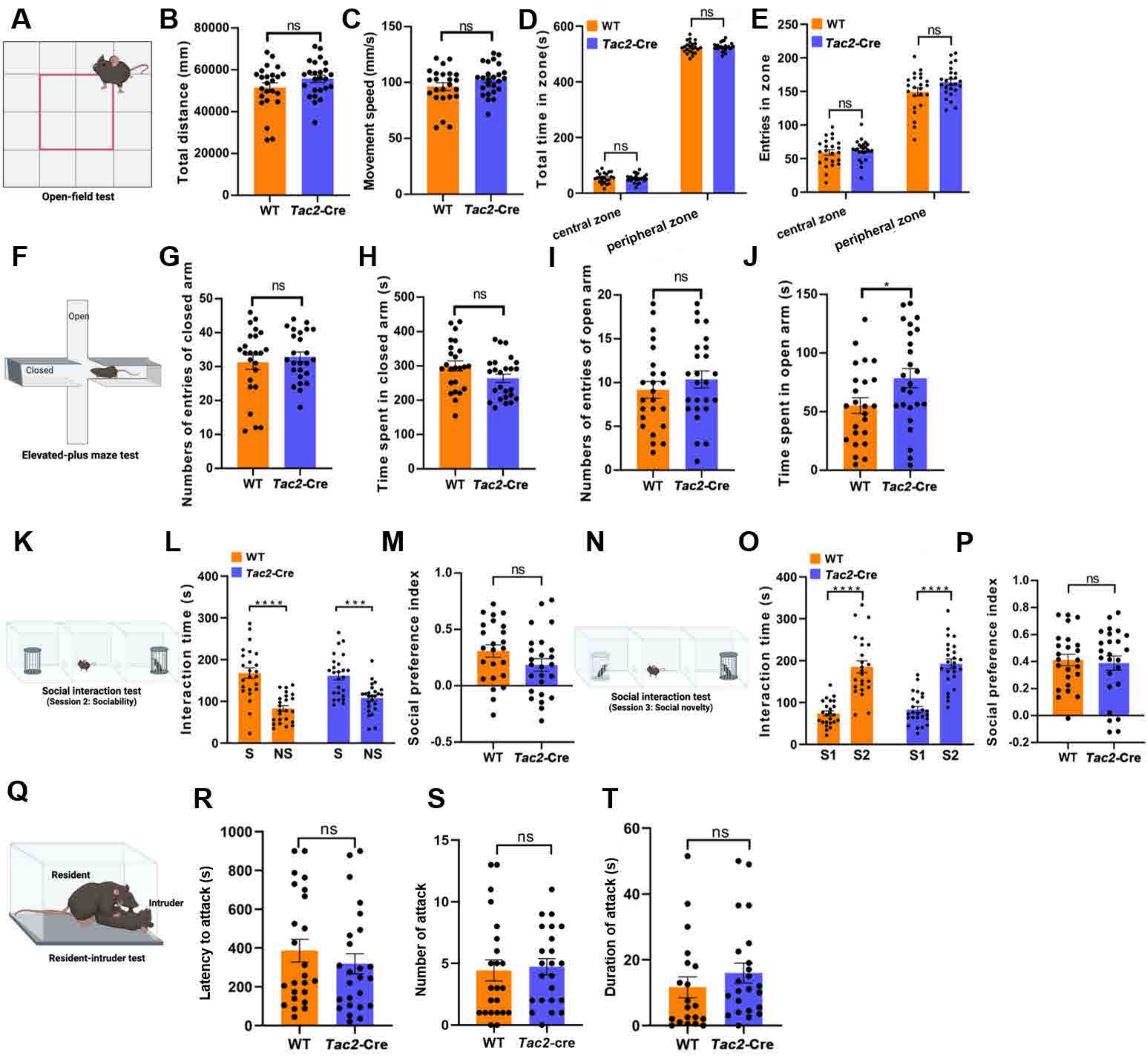
A
Open-field test
B
Total distance (mm)
80000
60000
40000
20000
0
ns
WT
Tac2-Cre
C
Movement speed (mm/s)
150
100
50
0
ns
WT
Tac2-Cre
D
WT
Tac2-Cre
Total time in zone (s)
600
400
200
0
ns
ns
central zone
peripheral zone
E
WT
Tac2-Cre
Entries in zone
250
200
150
100
50
0
ns
ns
central zone
peripheral zone
F
Open
Closed
Elevated-plus maze test
G
Numbers of entries of closed arm
50
40
30
20
10
0
ns
WT
Tac2-Cre
H
Time spent in closed arm (s)
500
400
300
200
100
0
ns
WT
Tac2-Cre
I
Numbers of entries of open arm
20
15
10
5
0
ns
WT
Tac2-Cre
J
Time spent in open arm (s)
150
100
50
0
*
WT
Tac2-Cre
K
Social interaction test
(Session 2: Sociability)
L
WT
Tac2-Cre
Interaction time (s)
400
300
200
100
0
****
***
S
NS
S
NS
M
Social preference index
1.0
0.5
0.0
-0.5
ns
WT
Tac2-Cre
N
Social interaction test
(Session 3: Social novelty)
O
WT
Tac2-Cre
Interaction time (s)
400
300
200
100
0
****
****
S1
S2
S1
S2
P
Social preference index
1.0
0.8
0.6
0.4
0.2
0.0
-0.2
ns
WT
Tac2-Cre
Q
Resident
Intruder
Resident-intruder test
R
Latency to attack (s)
1000
800
600
400
200
0
ns
WT
Tac2-Cre
S
Number of attack
15
10
5
0
ns
WT
Tac2-cre
T
Duration of attack (s)
60
40
20
0
ns
WT
Tac2-Cre

Figure S6

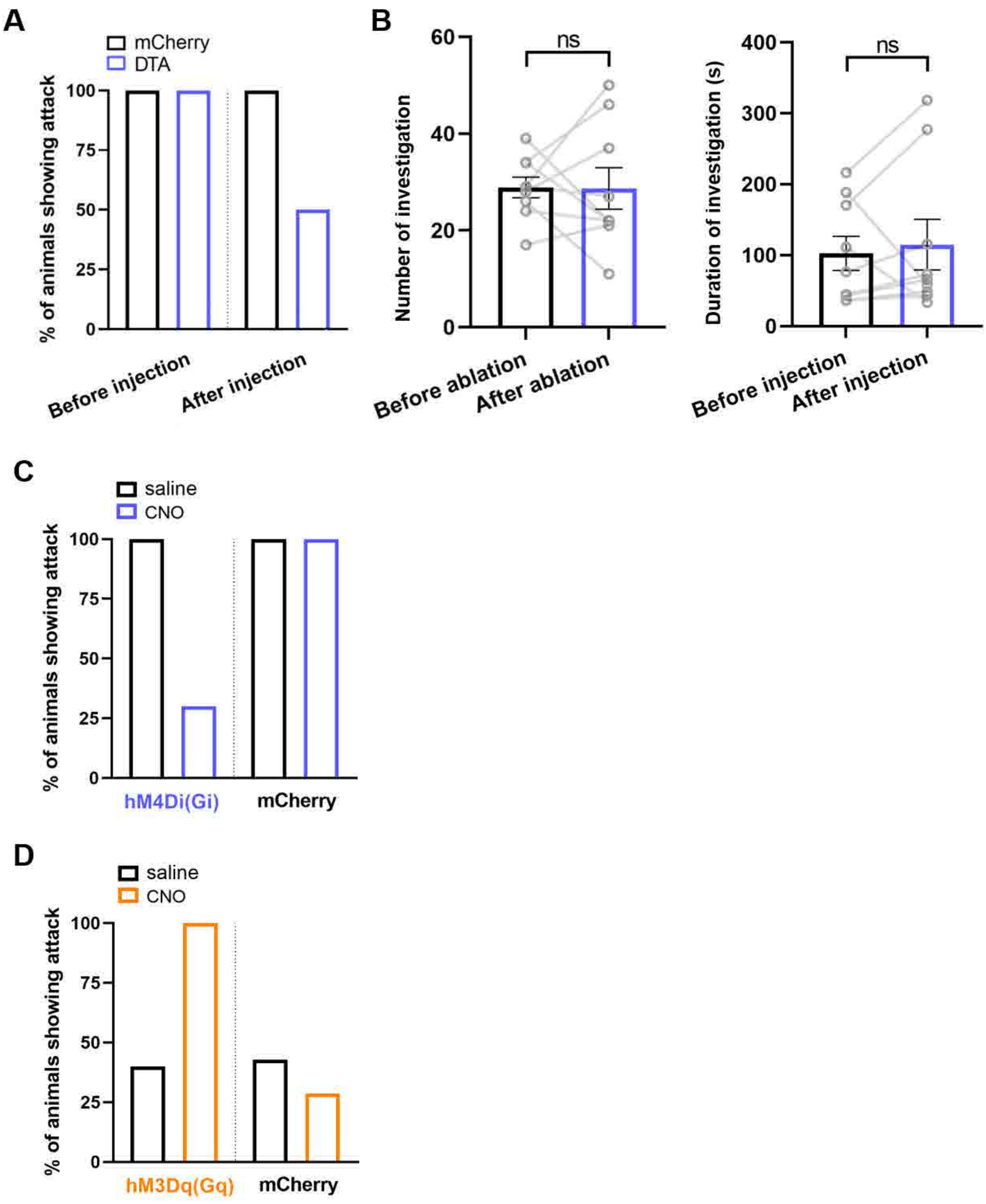
A
mCherry
DTA
% of animals showing attack
100
75
50
25
0
Before injection
After injection
B
Number of investigation
60
40
20
0
ns
Before ablation
After ablation
Duration of investigation (s)
400
300
200
100
0
ns
Before injection
After injection
C
saline
CNO
% of animals showing attack
100
75
50
25
0
hM4Di(Gi)
mCherry
D
saline
CNO
% of animals showing attack
100
75
50
25
0
hM3Dq(Gq)
mCherry

Figure S7

hM4Di

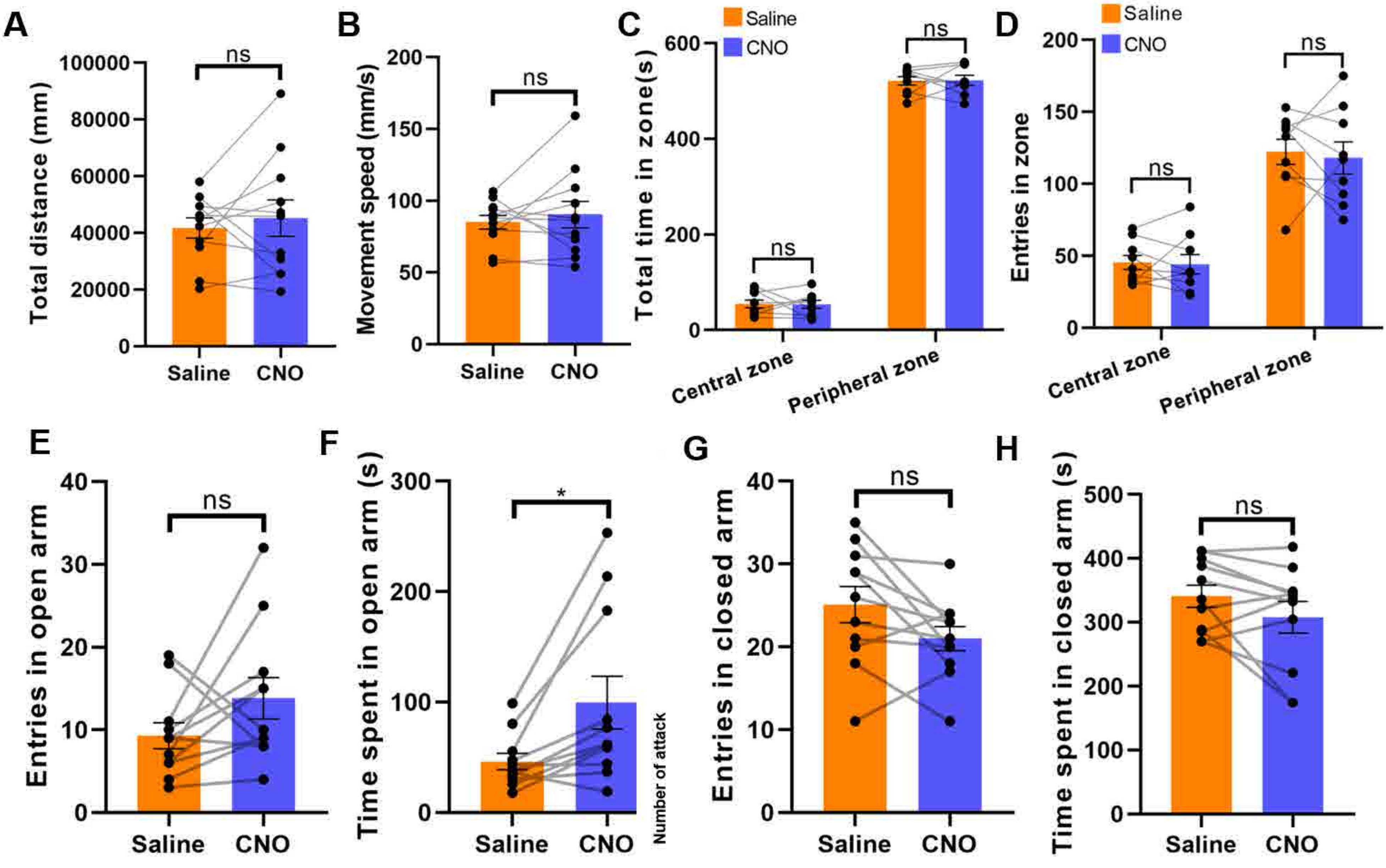

Figure S8

hM3Dq

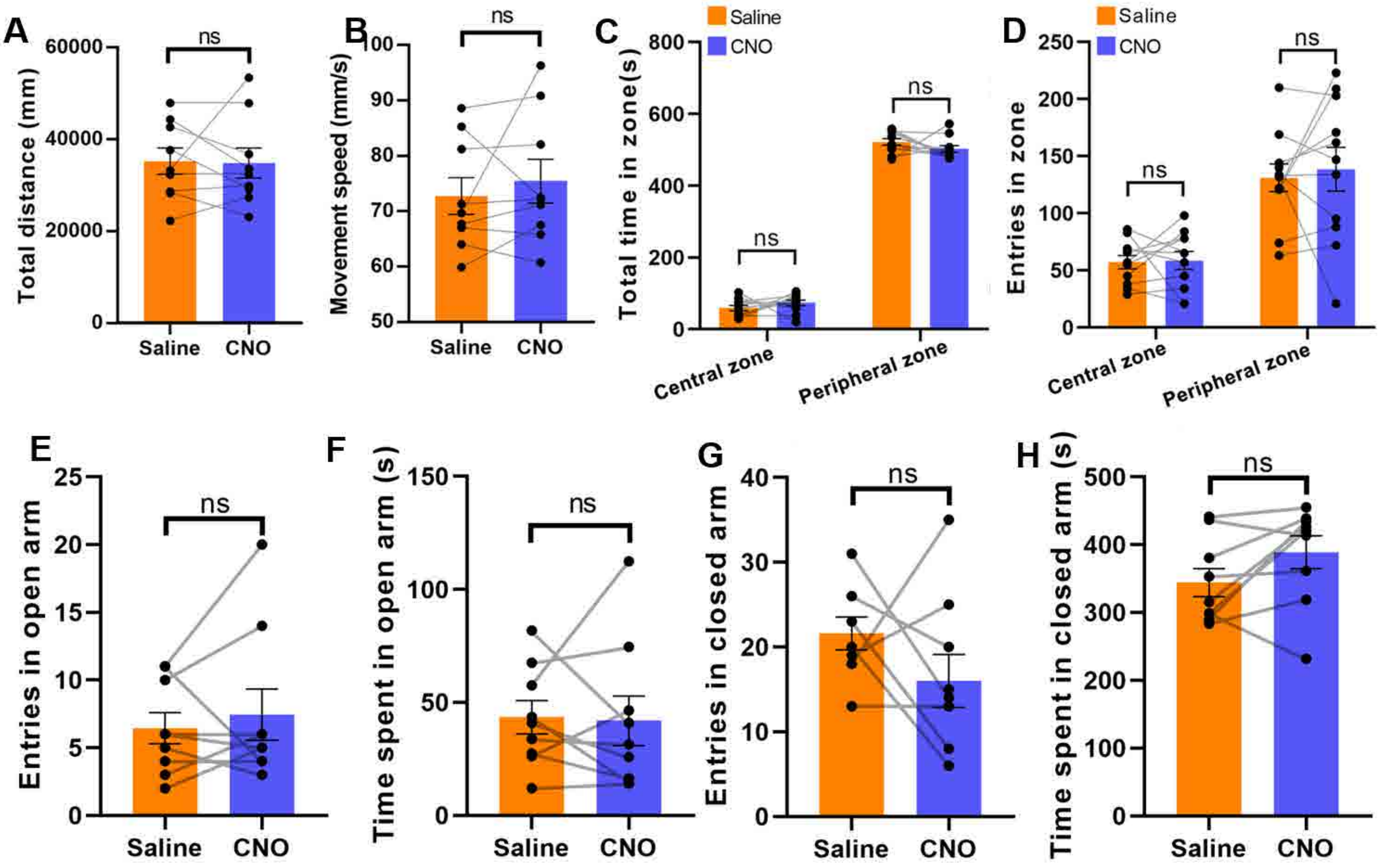

Figure S9

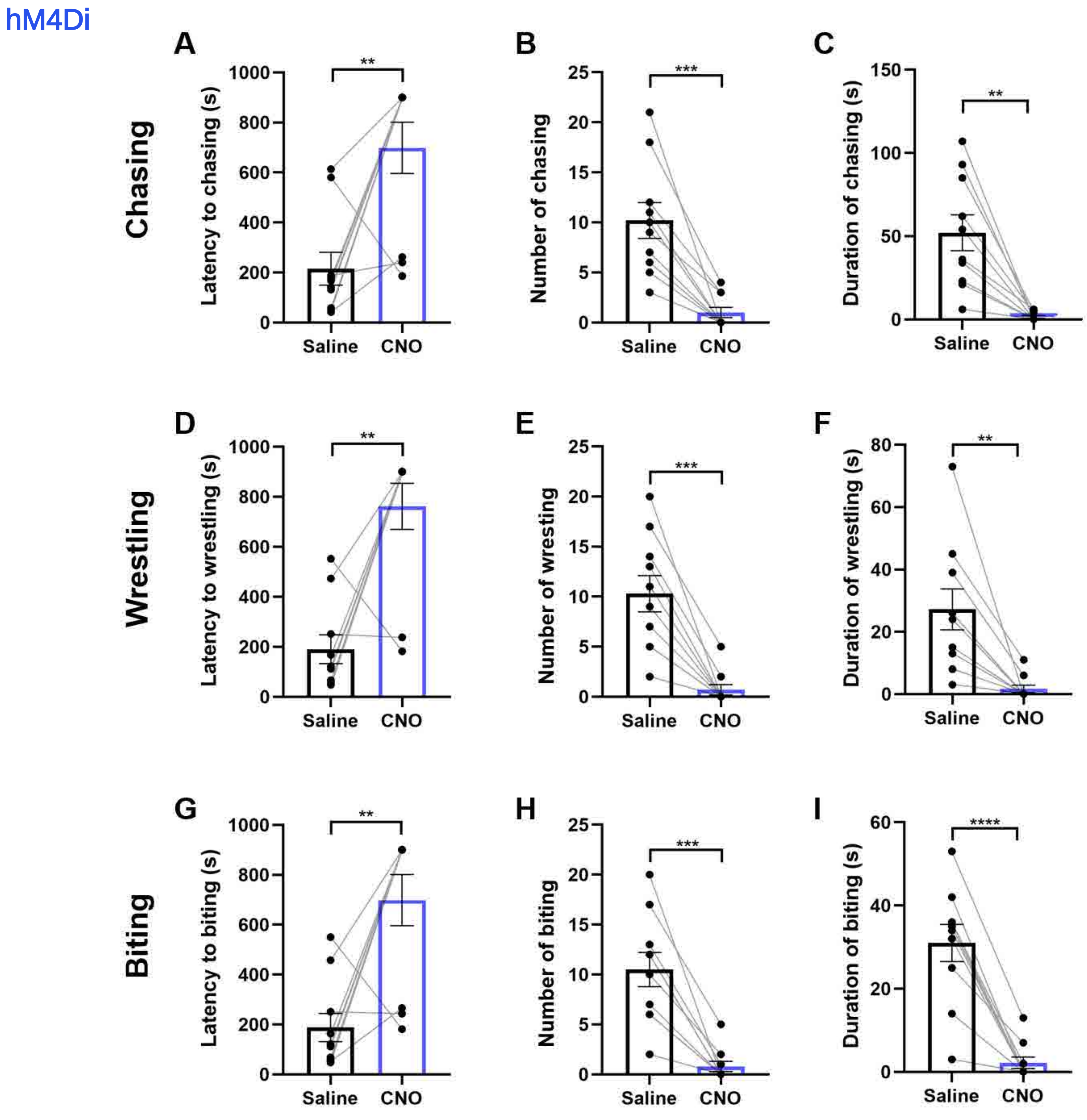
hM4Di
Chasing
Wrestling
Biting
A
B
C
D
E
F
G
H
I
Latency to chasing (s)
Number of chasing
Duration of chasing (s)
Latency to wrestling (s)
Number of wresting
Duration of wrestling (s)
Latency to biting (s)
Number of biting
Duration of biting (s)
Saline
CNO
**
***
****

Figure S10

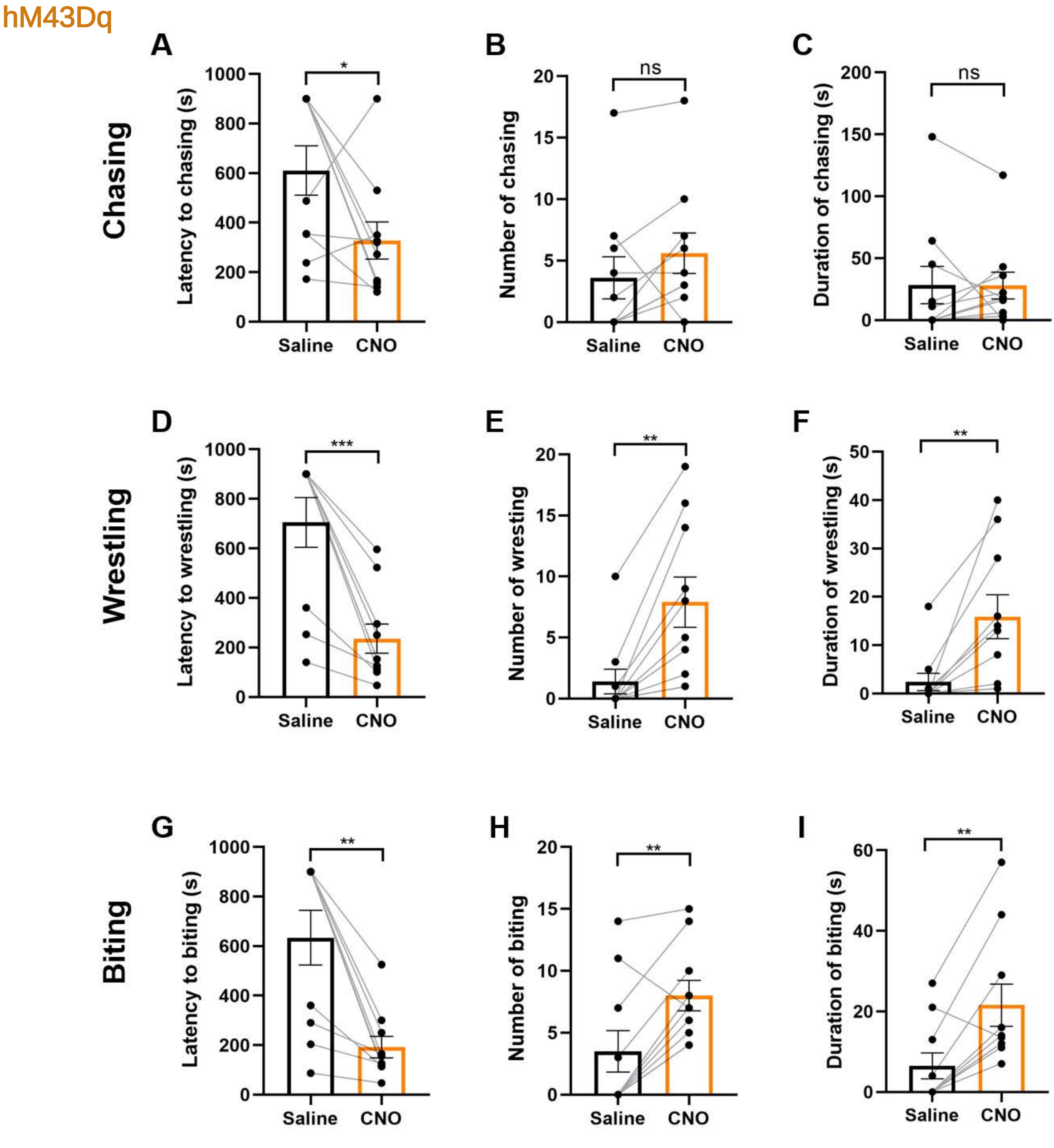
hM43Dq
A
B
C
D
E
F
G
H
I
Chasing
Wrestling
Biting
Latency to chasing (s)
Number of chasing
Duration of chasing (s)
Latency to wrestling (s)
Number of wresting
Duration of wrestling (s)
Latency to biting (s)
Number of biting
Duration of biting (s)
*
ns
***
**
Saline
CNO

Figure S11

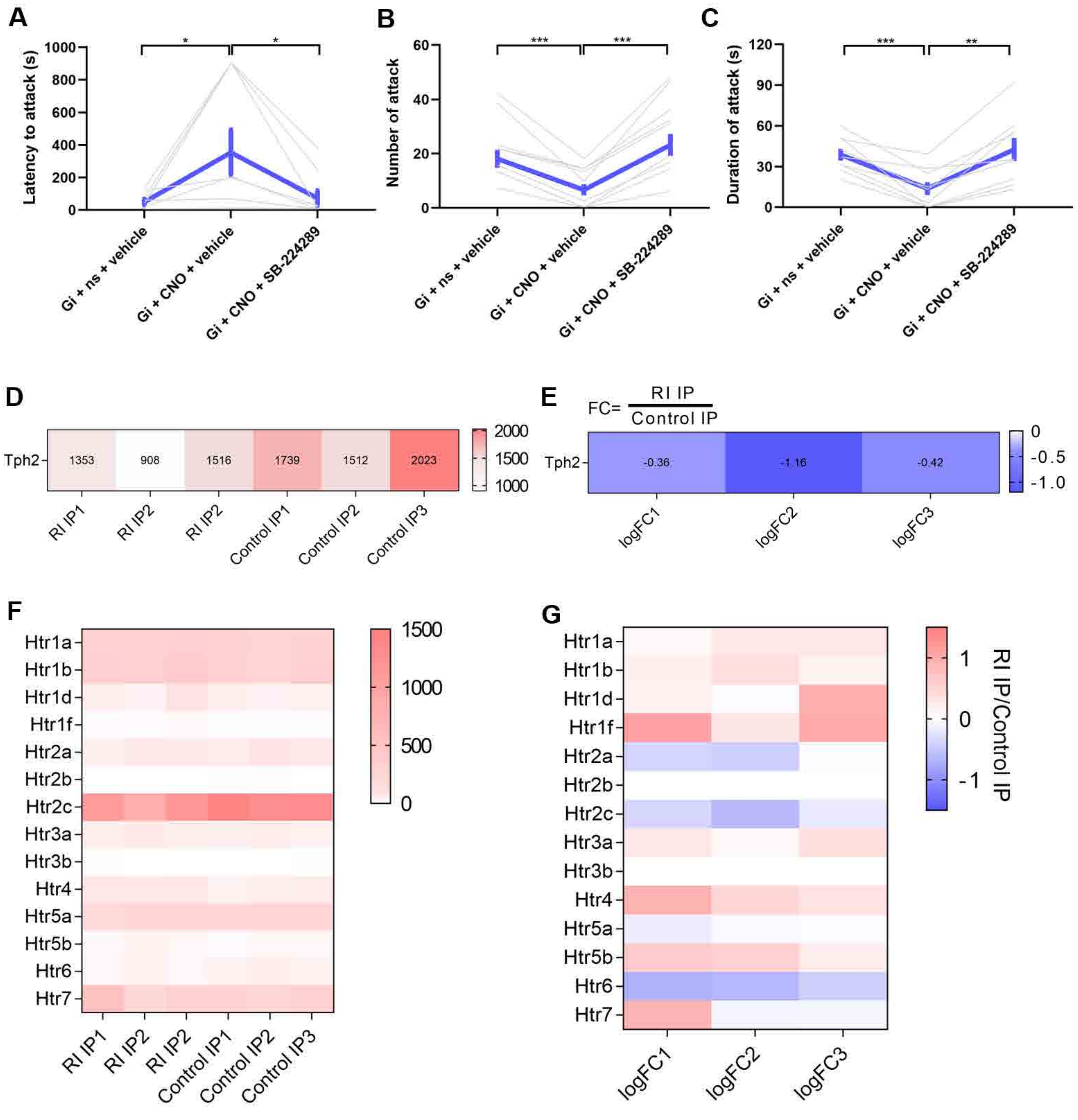
A
Latency to attack (s)
1000
800
600
400
200
0
*
*
Gi + ns + vehicle
Gi + CNO + vehicle
Gi + CNO + SB-224289
B
Number of attack
60
40
20
0
***
***
Gi + ns + vehicle
Gi + CNO + vehicle
Gi + CNO + SB-224289
C
Duration of attack (s)
120
90
60
30
0
***
**
Gi + ns + vehicle
Gi + CNO + vehicle
Gi + CNO + SB-224289
D
Tph2
1353
908
1516
1739
1512
2023
2000
1500
1000
RI IP1
RI IP2
RI IP2
Control IP1
Control IP2
Control IP3
E
FC= RI IP / Control IP
Tph2
-0.36
-1.16
-0.42
0
-0.5
-1.0
logFC1
logFC2
logFC3
F
Htr1a
Htr1b
Htr1d
Htr1f
Htr2a
Htr2b
Htr2c
Htr3a
Htr3b
Htr4
Htr5a
Htr5b
Htr6
Htr7
1500
1000
500
0
RI IP1
RI IP2
RI IP2
Control IP1
Control IP2
Control IP3
G
Htr1a
Htr1b
Htr1d
Htr1f
Htr2a
Htr2b
Htr2c
Htr3a
Htr3b
Htr4
Htr5a
Htr5b
Htr6
Htr7
1
0
-1
RI IP/Control IP
logFC1
logFC2
logFC3
